# Astaxanthin stimulates mitochondrial biogenesis in insulin resistant muscle via activation of AMPK pathway

**DOI:** 10.1002/jcsm.12530

**Published:** 2020-01-31

**Authors:** Yasuhiro Nishida, Allah Nawaz, Tomonobu Kado, Akiko Takikawa, Yoshiko Igarashi, Yasuhiro Onogi, Tsutomu Wada, Toshiyasu Sasaoka, Seiji Yamamoto, Masakiyo Sasahara, Johji Imura, Kumpei Tokuyama, Isao Usui, Takashi Nakagawa, Shiho Fujisaka, Yagi Kunimasa, Kazuyuki Tobe

**Affiliations:** ^1^ First Department of Internal Medicine University of Toyama Toyama Japan; ^2^ Fuji Chemical Industries, Co., Ltd. Toyama Japan; ^3^ Department of Metabolism and Nutrition University of Toyama Toyama Japan; ^4^ Department of Clinical Pharmacology University of Toyama Toyama Japan; ^5^ Department of Pathology University of Toyama Toyama Japan; ^6^ Department of Diagnostic Pathology University of Toyama Toyama Japan; ^7^ Doctoral Program in Sports Medicine, Graduate School of Comprehensive Human Sciences University of Tsukuba Tsukuba Japan; ^8^ Department of Endocrinology and Metabolism Dokkyo Medical University Tochigi Japan

**Keywords:** Astaxanthin, Mitchondrial biogenesis, Exercise‐endurance, Skeletal muscle remodeling, AMPK activation, insulin resistance

## Abstract

**Background:**

Skeletal muscle is mainly responsible for insulin‐stimulated glucose disposal. Dysfunction in skeletal muscle metabolism especially during obesity contributes to the insulin resistance. Astaxanthin (AX), a natural antioxidant, has been shown to ameliorate hepatic insulin resistance in obese mice. However, its effects in skeletal muscle are poorly understood. The current study aimed to investigate the molecular target of AX in ameliorating skeletal muscle insulin resistance.

**Methods:**

We fed 6‐week‐old male C57BL/6J mice with normal chow (NC) or NC supplemented with AX (NC+AX) and high‐fat‐diet (HFD) or HFD supplemented with AX for 24 weeks. We determined the effect of AX on various parameters including insulin sensitivity, glucose uptake, inflammation, kinase signaling, gene expression, and mitochondrial function in muscle. We also determined energy metabolism in intact C2C12 cells treated with AX using the Seahorse XFe96 Extracellular Flux Analyzer and assessed the effect of AX on mitochondrial oxidative phosphorylation and mitochondrial biogenesis.

**Results:**

AX‐treated HFD mice showed improved metabolic status with significant reduction in blood glucose, serum total triglycerides, and cholesterol (*p*< 0.05). AX‐treated HFD mice also showed improved glucose metabolism by enhancing glucose incorporation into peripheral target tissues, such as the skeletal muscle, rather than by suppressing gluconeogenesis in the liver as shown by hyperinsulinemic–euglycemic clamp study. AX activated AMPK in the skeletal muscle of the HFD mice and upregulated the expressions of transcriptional factors and coactivator, thereby inducing mitochondrial remodeling, including increased mitochondrial oxidative phosphorylation component and free fatty acid metabolism. We also assessed the effects of AX on mitochondrial biogenesis in the siRNA‐mediated AMPK‐depleted C2C12 cells and showed that the effect of AX was lost in the genetically AMPK‐depleted C2C12 cells. Collectively, AX treatment (i) significantly ameliorated insulin resistance and glucose intolerance through regulation of AMPK activation in the muscle, (ii) stimulated mitochondrial biogenesis in the muscle, (iii) enhanced exercise tolerance and exercise‐induced fatty acid metabolism, and (iv) exerted antiinflammatory effects via its antioxidant activity in adipose tissue.

**Conclusions:**

We concluded that AX treatment stimulated mitochondrial biogenesis and significantly ameliorated insulin resistance through activation of AMPK pathway in the skeletal muscle.

## 1 Introduction

Recent studies have shown that the increasing prevalence of obesity and associated comorbidities such as type 2 diabetes, cardiovascular disease, and certain cancers represents a major threat to public health driven by skeletal muscle.^1^ Previous reports showed that insulin resistance in skeletal muscle is main characteristic feature of type 2 diabetes. Several transcriptional factors including PPARα, PPARδ and PPARγ in the skeletal muscle involved in enhancing insulin sensitivity, glucose tolerance, energy expenditure, and lipid metabolism.^2^, ^3^, ^4^, ^5^, ^6^, ^7^, ^8^ Activation of these transcriptional factors enhanced mitochondrial biogenesis leads to dramatically increased endurance, ameliorated insulin resistance in obesity, and type 2 diabetes.^9^, ^10^, ^11^, ^12^, ^13^ It has been known that mitochondrial dysfunction and cellular oxidative stress induced by excess energy states and elevations in circulating free fatty acid (FFA) are associated with insulin resistance in skeletal muscle.^14^, ^15^, ^16^, ^17^, ^18^, ^19^, ^20^ Therefore, in order to improve skeletal muscle insulin resistance and energy expenditure, it is important to stimulate mitochondrial biogenesis to preserve mitochondrial pool and to increase mitochondrial oxidative phosphorylation and FFA metabolism.

Carotenoids are widely known for their antioxidant activities, and it has been shown that AX, a xanthophyll carotenoid, possesses a particularly high antioxidant capacity, as it has more conjugated double bonds than many other carotenoids.^21^ AX has also been demonstrated to ameliorate hepatic insulin resistance in obese animals 22, 23, 24 and to partially improve metabolic parameters in obese humans.^25^, ^26^ However, no systematic investigation has been conducted yet to clarify whether AX improves the insulin sensitivity in skeletal muscle of obese mice. In fact, we previously showed that AX positively regulated insulin signaling and enhanced glucose uptake in differentiated L6 myotubes.^27^ On the other hand, AX has been shown to be of potential therapeutic usefulness for the prevention and treatment of insulin resistance in the liver through reducing oxidative stress and inflammation, and thereby to be useful for the prevention of nonalcoholic fatty liver disease, nonalcoholic steatohepatitis, liver cirrhosis, and hepatocellular carcinoma.^28^, ^29^, ^30^, ^31^ However, it is still unclear whether these effects of AX are attributable to its antiinflammatory effects mediated by its antioxidant activity. Thus, we examined the mechanisms underlying the amelioration of insulin resistance in skeletal muscle by AX both *in vivo* and *in vitro*.

## 2 Methods

### 2.1 Reagents

Cell culture reagents were purchased from Invitrogen (Carlsbad, CA); commerciall\y available Astaxanthin (AX) powder was purchased from Fuji Chemical Industries USA, Inc. (P2AF, Burlington, NJ); anti‐Akt, anti‐p‐Akt (S473), anti‐AMPK, anti‐p‐AMPK (T172), anti‐p‐Acetyl‐CoA carboxylase (ACC), anti‐ACC, and anti‐β‐Actin were purchased from Cell Signaling Technology (Danvers, MA); anti‐Sirt1 was purchased from Millipore (Billerica MA). Anti‐PGC‐1 and total OXPHOS rodent WB antibody cocktail were purchased from Abcam (Cambridge, MA). 5‐Aminoimidazole‐4‐carboxamide ribonucleotide (AICAR), AMPK agonist, was purchased from AdipoGen Life Science (AG‐CR1‐0061‐M050). Horse radish peroxidase‐conjugated antirat or antirabbit secondary antibody and the ECL western blot detection reagent were obtained from GE Healthcare (Buckinghamshire, UK). All other reagents were purchased from Sigma‐Aldrich.

### 2.2 Animals

Five‐week‐old male C57BL/6J mice were purchased from Sankyo Laboratory Service (Tokyo, Japan). All animals were housed under a 12‐h light/12‐h dark cycle and allowed free access to food and water. The regular diet (normal chow [NC]; D12450B) and 60% high‐fat diet (HFD; D12492) and the AX pre‐mixed diets (final AX content using commercially available AX powder was 0.02%) were purchased from Research Diets Inc. (New Brunswick, NJ). One week after habitation, from 6 week old, they were started fed an each diet. Then, 8, 16, or 24 weeks later, the mice were sacrificed under anesthesia by intraperitoneal injection. The time schedules are shown in detail in Figure S1. The animal care policies and procedures for the experiments were approved by the Animal Experiment Committee at the University of Toyama.

### 2.3 Cell culture

C2C12 cells (American Type Culture Collection; ATCC® CRL‐1772™, Manassas, Virginia) were cultured as described previously,^32^ with some modification. Briefly, C2C12 cells were grown to confluency (60–70%) in growth medium containing low glucose Dulbecco's Modified Eagle's Medium (DMEM, Thermo Fisher Scientific) with 10% fetal bovine serum (Gibco™ 10437‐028) without antibiotics at 37°C in 5% CO_2_. Two‐day postconfluency, differentiation was induced by using differentiation medium (DM) containing low glucose DMEM with 2% horse serum (Invitrogen). The DM was replenished every day. Three or 4 days after differentiation, the C2C12 cells were treated with AX (1, 5, 25, or 50μM), AICAR 500μM or others regents and vehicle (0.2% dimethyl sulfoxide) for specified time.

### 2.4 Real‐time polymerase chain reaction

Tissues for real‐time polymerase chain reaction (RT‐PCR) were collected and preserved in RNA later solution from Ambion (Austin, TX), in accordance with the manufacturer's instructions. All tissues, except skeletal muscle, were lysed with buffer RLT in the RNeasy kit (Qiagen, Hilden, Germany). For skeletal muscle, approximately 100 mg of the tissue was minced well and lysed with 1 mL of Isogen (Nippon gene, Toyama, Japan), followed by addition of 20% v/v of chloroform. After centrifugation, water phase was used for further processing. RNeasy kit was applied to the lysate to extract and purify RNA. Total RNA was reverse‐transcribed using the TaKaRa PrimeScript RNA Kit (TaKaRa Bio, Shiga, Japan), in accordance with the manufacturer's instructions. Quantitative PCR of the genes was performed using the TaqMan method (1 cycle at 50°C for 2 min, at 95°C for 10 min, and 40 cycles at 95 °C for 15 s, at 60°C for 1 min) using premade primer sets. The relative mRNA expression levels were calculated using the *ΔΔ*Ct and normalized to the mRNA levels of *β‐actin* or *Tf2b*. The SYBR Green thermal cycling conditions were 1 cycle at 95°C for 30 s, and 45 cycles at 95°C for 10 s and at 60°C for 20 s. The relative mRNA expression levels were calculated using the standard curve method and normalized to the mRNA levels of *β‐actin* or *Tf2b*. All mRNA slicing variants of the *Pgc‐1α* gene were measured by a previously reported method.^33^, ^34^ Complete list of mouse SYBR green primers is given in Table S1.

### 2.5 Western blotting

Tissues for the western blot analysis were quickly frozen in liquid nitrogen and preserved at −80°C until the analysis. The western blot analysis was performed as described previously.^35^ Briefly, the tissues for western blotting were homogenized in lysis buffer containing 25 mM Tris–HCl (pH 7.4), 10 mM Na_3_VO_4_, 100 mM NaF, 50 mM Na_4_P2O_7_, 10 mM EDTA, 0.2% leupeptin (5 mg/mL), 0.5% aprotinin (5 mg/mL), 2 mM phenylmethylsulfonyl fluoride, and 1% Nonidet P‐40, using a Multi‐Beads Shocker cell disrupter (Yasui Kikai Corporation, Osaka, Japan). The lysates were centrifuged to remove any insoluble materials and mixed with loading buffer before protein denaturation by boiling at 95°C for 3–5 min. For OXPHOS proteins, the lysis buffer was changed to RIPA buffer and heat denaturation was not applied. The samples were incubated at 37°C for 5 min. The protein content in all the samples was adjusted to a concentration of 1μg/μL. The protein lysates were run on 7.5 or 10% separating gels and transferred to PVDF Immobilon‐P transfer membranes (Millipore, Billerica MA). The membranes were incubated overnight at 4°C with the primary antibody (1:500–2000 dilution) and for 2 h at room temperature with the secondary antibody (1:2000 dilution), before being subjected to a western blot detection reagent immediately before image development.

### 2.6 Glucose tolerance test and insulin tolerance test

For the intraperitoneal glucose tolerance test, the mice were fasted for 18 h and were administered an intraperitoneal injection of glucose; 1 mg/g body weight (BW). For the intraperitoneal insulin tolerance test, mice fasted for 2–3 h and were administered an intraperitoneal injection of human insulin (0.8 units/kg BW for the mice fed NC and 1.2 units/kg BW for the mice fed HFD.^35^, ^36^, ^37^, ^38^ Blood samples were then collected from the tail vein at 0, 15, 30, 45, 60, 90, and 120 min after the injection for glucose/insulin measurement. The blood glucose levels obtained from the tail tip of the mice were measured using STAT STRIP Express 900 (Nova Biomedical, Waltham MA), and the serum insulin levels were determined using the Mouse Insulin ELISA KIT (Shibayagi, Shibukawa, Japan).

### 2.7 Hyperinsulinemic–euglycemic clamp study

The clamp study was performed on 8‐ to 10‐week‐old or 20‐ to 24‐week old HFD or HFD+AX fed mice, which showed BWs in the same range, under a conscious and unstressed condition, after the animals had been denied access to food for 6 h, as described previously.^35^ A primed‐continuous infusion of insulin (Humulin R; Lilly) was given at the rate of 10.0 mU/kg/min to the HFD mice, and the blood glucose concentration (4:1) ratio, monitored every 5 min, was maintained at ~120 mg/dl for 120 min by administration of glucose (50% glucose enriched to ~20% with 50% D_2_‐glucose (Santa Cruz Biotechnology, Dallas, USA). Blood samples were collected at 0, 90, 105, and 120 min for determination of the rate of glucose disappearance (Rd), and hepatic glucose production or endogenous glucose production was calculated as the difference between the Rd and exogenous glucose infusion rate.^35^

### 2.8 Exercise tolerance test

An exercise tolerance test was performed by a procedure reported previously by others, with slight modification.^39^ In brief, the test was performed after mice had been denied access to food for 2 h, using a multispeed belt treadmill with a stimulus device consisting of a shock grid attached to the rear end of the belt (MK‐690, Muromachi Kikai Co., Ltd.). Exercise training for the mice was conducted as described in a previous report,^40^ with slight modification. In brief, the training was started at 10 m/min for 10 min/day for 3 days. Then, over a 3‐week period, the intensity was gradually increased to 28 m/min, while maintaining the duration 10 min, over a period of 8 weeks. All animals were acclimatized to the test using a habituation protocol on the day prior to the running test. All the mice were made to run at 15 m/min for 10 min on a 5′ incline. The endurance test and glucose tolerance test were performed after 8 weeks on each diet.

For the actual endurance test, the mice were made to run at 15 m/min on a 5′ incline. The speed and incline were increased progressively by the method described by Lagouge M. et al.^39^ The distance run and the number of shocks obtained over 5‐min intervals were recorded, and a mouse was considered as having become exhausted and removed from the treadmill when it received approximately 100 shocks with a period of 5 min.

### 2.9 Cellular metabolic rate

Analysis of energy metabolism in intact C2C12 cells was performed using the Seahorse XFe96 Extracellular Flux Analyzer (Seahorse Bioscience/Agilent, Santa Clara, CA). C2C12 cells were seeded at 10 000 cells per well into 96‐well XF plates. After allowing differentiation for 4 days, further differentiation was induced by addition of DM, followed by addition of AX or vehicle and incubation for 24 h. The C2C12 cells were then shifted to a sodium bicarbonate‐free medium and incubated for 1h at 37°C in a non‐CO_2_ incubator. Cellular oxygen consumption rate measurements were obtained at the baseline and following injection of oligomycin (1 μM), FCCP (1 μM), and antimycin A plus rotenone (AA/ROT, 1 μM). For measurement of the extracellular acidification rate (ECAR), the assay medium was supplemented with glutamine (1 mM). The measurements were performed at the baseline and after injection of glucose (10 mM), oligomycin (1 μM), and 2‐DG (100 mM). At least five independent basal measurements were performed for each assay. Three measurements were performed after each injection, with each measurement consisting of a 10‐s mixing period and 3‐min measurement period, in accordance with the manufacturer's instructions.

To evaluate oxidation of exogenous FFAs, the C2C12 cells were incubated in substrate‐limited medium in the presence or absence of AX for 24 h. Thereafter, the C2C12 cells were shifted to FAO assay medium containing XF Palmitate‐BSA FAO Substrate/BSA control (p/n 102720‐100, Agilent) and 40 μM of etomoxir, a CPT‐1 (carnitine palmitoyl transferase‐1) inhibitor, followed by determination of the oxygen consumption rate. The maximal respiratory rate due to utilization of exogenous FFAs was calculated in accordance with the manufacturer's instructions. (#5991‐7149EN).

### 2.10 AMPK small interfering RNA transfection

C2C12 cells were seeded in growth medium without antibiotics for 18‐24h, then differentiation was started by adding DM. 48h after the onset of differentiation, C2C12 cells were cultured with small interfering RNA (siRNA) duplexes specific for mouse AMPKα1/2 (sc‐45313, Santa Cruz Biotechnology) or non‐target control (NTC) siRNA (sc‐36869, Santa Cruz Biotechnology) using the transfection reagents (sc‐29528, Santa Cruz Biotechnology) for 6‐7h according to the manufacturer's instructions. After transfection, fresh DM was added for next 24 h. The medium was replaced with fresh DM for additional 24 h. Then transfected C2C12 cells were treated with AX (50 μM) or AICAR (500 μM) or vehicle for specified time. The cells were collected in Isogen and subjected to RNA extraction.

### 2.11 Oxidative stress analysis

Oxidative stress was evaluated by measuring the production of malondialdehyde and thiobarbituric acid‐reactive substances (TBARS) in frozen tissue samples. TBARS assay was performed using a malondialdehyde assay kit (Northwest Life Science Specialties, LLC., Vancouver, WA), in accordance with the manufacturer's instructions.

### 2.12 Measurement of blood pressure, triglyceride, and total cholesterol

Systolic blood pressure was measured in conscious mice by the tail‐cuff method (Muromachi Kikai Co., Ltd). Triglyceride and total cholesterol (Chol) were measured in mouse serum (18h fasted mice) by LabAssay Triglyceride (Wako) and LabAssay Cholesterol (Wako), respectively, in accordance with the manufacture's instruction.

### 2.13 Statistical analysis

Statistical analyses were performed using an unpaired Student's *t* test or analysis of variance (ANOVA), with Dunnett's posttest or Bonferroni's correction. Differences at *p < 0.05, **p < 0.01, and ***p < 0.001 were considered as being statistically significant. The results are presented as the means ± SEM.

## 3 Results

### 3.1 AX prevented the development of metabolic syndrome in HFD mice

**Figure 1:**
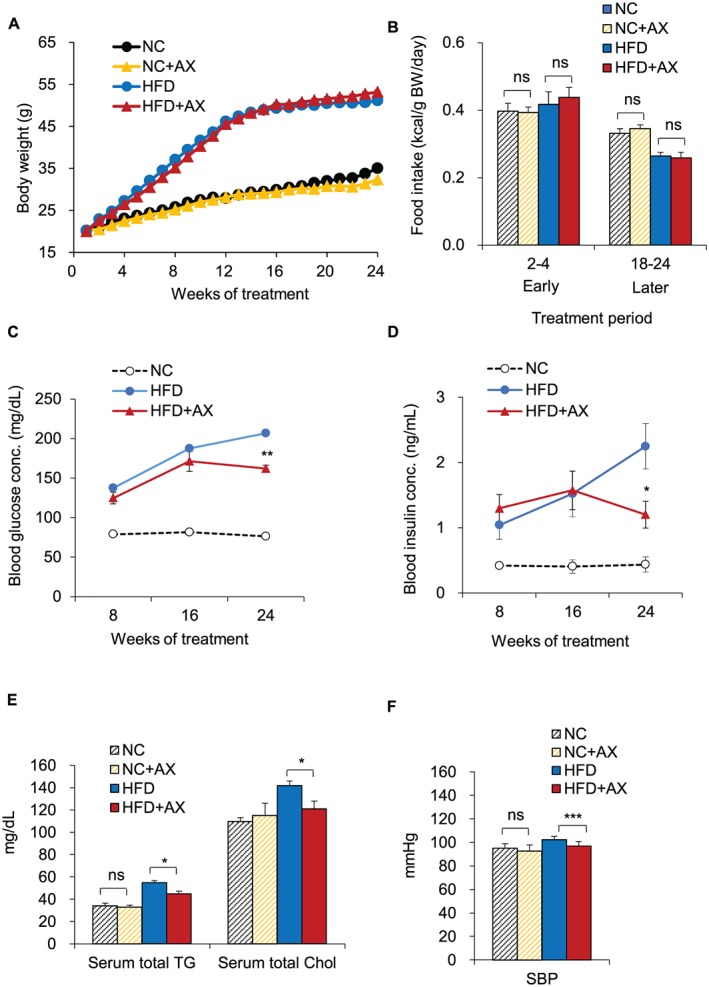
Astaxanthin (AX) improved the metabolic status in high‐fat‐diet (HFD) C57BL/6J mice. (A) Body weights of C57BL/6J mice fed HFD alone (HFD group, *n* = 10) or HFD supplemented with AX (HFD+AX group, *n* = 10). Six‐week‐old male mice were fed HFD for 24 weeks. (B) Average daily food intake in the early (2–4 weeks) and late (20–24 weeks) experimental period (*n* = 10 in each group). (C,D) Changes in the blood levels of glucose (C), insulin (D) in the AX‐treated HFD mice compared with control HFD and NC mice (*n* = 8 per group). (E) Changes in serum total triglyceride (TG) and total cholesterol in the AX‐treated HFD and NC mice compared with non‐AX‐treated HFD and NC mice (*n* = 5‐8 per group). (F) Changes of the blood pressure in the AX‐treated HFD and NC mice compared with non‐AX‐treated HFD and NC mice (*n* = 6 per group). All values are presented as the means ± S.E.M. *p < 0.05, **p < 0.01, ***p < 0.001, ns; non‐significant. (HFD vs. HFD+AX) (NC vs. NC+AX). Statistical tests were performed as follows: (A) two‐way repeated‐measures ANOVA, (B,E,F) Student's *t* test, and (C,D) two‐way repeated‐measures ANOVA.

To understand the role of AX in the regulation of insulin sensitivity in the skeletal muscle, we reared 6‐week‐old C57BL/6J mice on either NC or a HFD containing 60% energy as fat (NC mice and HFD mice, respectively). The NC or HFD administered to the mice was either mixed or not mixed with AX (Figure S1). AX treatment had no effect on the BW or food intake in either the NC or the HFD mice (Figures 1A and 1B). We found significant decrease in the fasting blood glucose (Figure 1C), insulin levels (Figure 1D) after 24 weeks, but not after 8 or 16 weeks, of AX treatment in the HFD mice, while AX treatment had no effect on the fasting blood glucose level and insulin levels in the NC mice treated with AX (Figures S2A and S2B). Significant decreases of HOMA‐R (Figure S2C) were observed after 24 weeks, but not after 8 or 16 weeks, of AX treatment in the HFD mice, while AX treatment had no effect on the insulin resistance index on the NC mice treated with AX (Figure S2D). Significant improvement of the HbA1c (Figure S2E) was also observed after 16 weeks and 24 weeks of AX treatment in HFD mice, while AX treatment had no effect on the HbA1c in NC mice (Figure S2F). We also observed significant reduction of the serum triglyceride and total cholesterol levels only after 24 weeks of AX treatment in the HFD mice (Figure 1E). AX treatment had no effect on the serum triglyceride and total cholesterol levels in the AX‐treated NC mice compared with NC control mice (Figure 1E). In addition, the HFD mice showed significant lowering of the systolic blood pressure after AX treatment (Figure 1F). On the other hand, AX treatment had no effect on the systolic blood pressure in the NC mice (Figure 1F). Altogether these data indicate that AX improved glucose metabolism in mice with diet‐induced obesity.

### 3.2 AX markedly ameliorated glucose intolerance and insulin resistance

**Figure 2:**
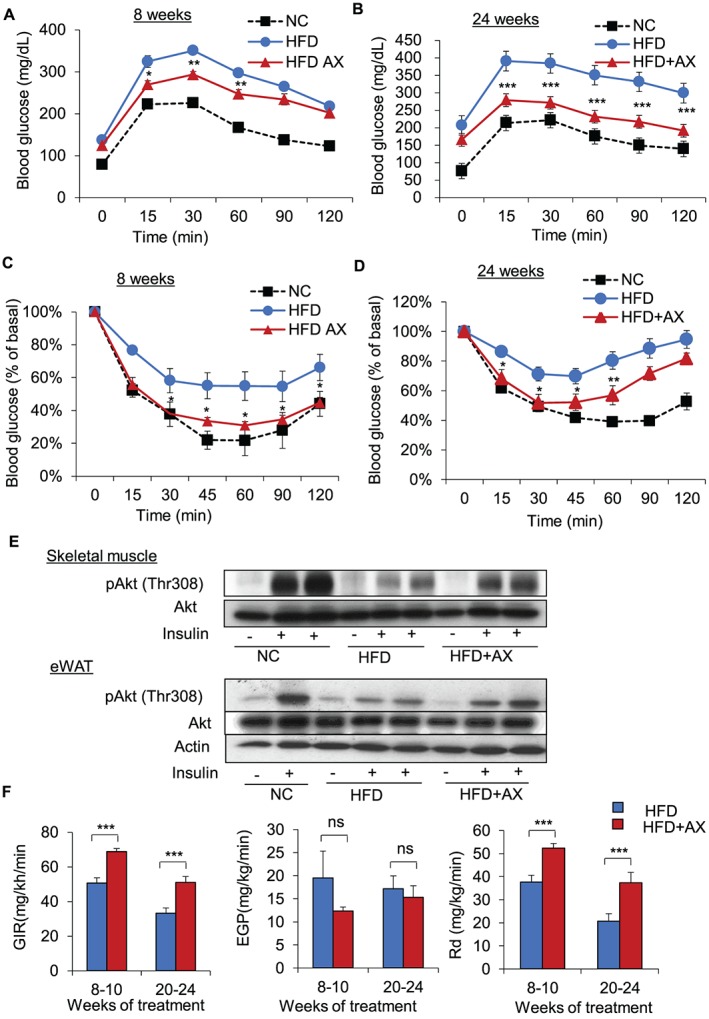
AX ameliorated the development of glucose intolerance and insulin resistance in lean and obese mice. (A,B) Intraperitoneal glucose tolerance test (IP‐GTT) and (C,D) Intraperitoneal insulin tolerance test (IP‐ITT) in the AX‐treated HFD mice compared with control HFD and NC mice for 8 (A,C) or 24 weeks (B,D) (*n* = 5–9 per group). (E) Western blot analysis of insulin‐induced phosphorylation of Akt in the skeletal muscle or epididymal white adipose tissue (eWAT). (F) Glucose infusion rate (*left*), endogenous glucose production (EGP) or HGP (*middle*), and the rates of glucose disposal (Rd) (*right*) during the hyperinsulinemic‐euglycemic clamp study in the mice fed HFD or HFD+AX for 8–10 or 20–24 weeks (*n* = 6‐9 per group). All values are represented as means ± S.E.M. *p < 0.05 (HFD vs. HFD+AX). Statistical tests were performed as follows: (A, B; IP‐GTT and C, D; IP‐ITT) two‐way repeated‐measures ANOVA, and (F) Student's *t*‐test.

To determine whether AX treatment affects glucose homeostasis, we compared the glucose tolerance and insulin sensitivity in AX‐treated NC and control NC mice as well as AX‐treated HFD mice and control HFD mice. We found that HFD‐induced glucose intolerance and insulin resistance was improved by AX‐treated HFD mice after 8 and 24 weeks of treatment (Figures 2A–2D). On the other hand, AX treatment had no effect on the glucose intolerance and insulin sensitivity in the NC mice (Figures S3A and S3B). Consistent with the above findings, AX treatment significant enhanced insulin‐stimulated phosphorylation of Akt in the skeletal muscle and adipose tissue of HFD‐fed mice (Figure 2E). To examine which of these mechanisms is responsible for the insulin‐sensitive phenotype, we performed hyperinsulinemic‐euglycemic clamp study. AX treatment enhanced the glucose infusion rate, while it had no effect on the insulin‐induced inhibition of hepatic glucose production, indicating that improved glucose metabolism in the skeletal muscle is responsible for the improved insulin sensitivity in the AX‐treated HFD mice (Figure 2F).

**Figure 3:**
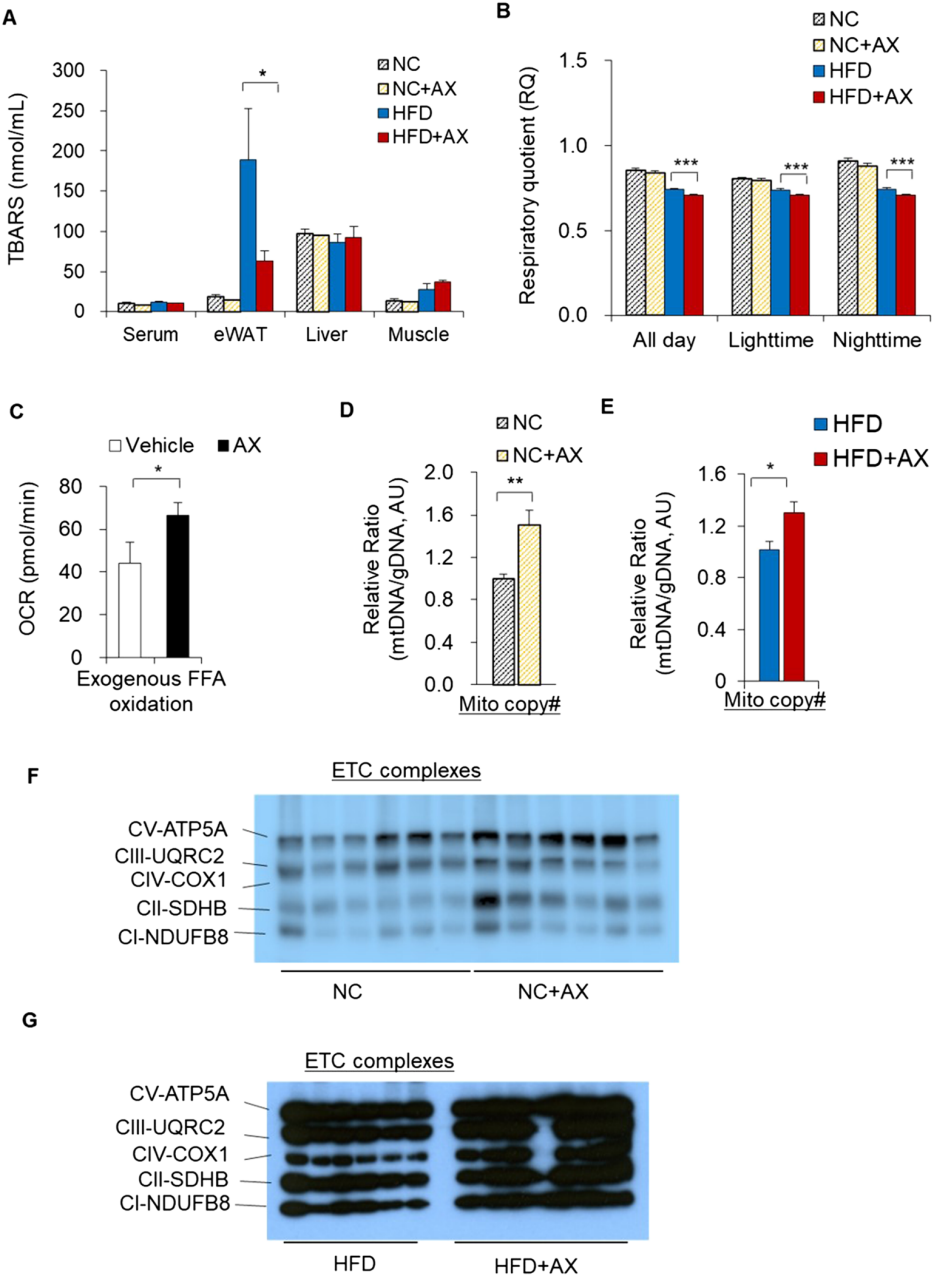
AX potentially enhanced the mitochondrial number and fatty acid utilization in skeletal muscle in HFD obese C57BL/6J mice. (A) AX partially reduced the oxidative stress in the adipose tissue of HFD obese C57BL/6J mice. Oxidative stress markers in the serum, skeletal muscle, eWAT, and the liver of AX‐treated HFD and NC mice compared with non‐AX‐treated HFD and NC mice. Oxidative stress was evaluated based on the production of malondialdehyde and TBARS (*n* = 3 in each group). (B) The respiratory quotient (RQ) in the mice fed NC, NC+AX, HFD, or HFD+AX for 8 weeks (*n* = 6 per group). (C) Exogenous free fatty acid (FFA) oxidation rates were measured in C2C12 cells treated with vehicle or 5 μM AX for 24 h, using oxygen consumption rate (OCR) as a readout with/without 40μM etomoxir and/or BSA‐Palmitate (*n* = 6 per group) using the XFe96 Extracellular Flux. (D,E) Relative mtDNA copy number in the gastrocnemius muscles of the C57BL/6J mice fed NC, NC+AX (D), HFD, or HFD+AX (E) (for 24 weeks (*n* = 6 per group). (F,G) Western blot analysis of mitochondrial electronic chain transport complexes (ETCs) in the C57BL/6J mice fed NC, NC+AX (F), HFD or HFD+AX (G) for 24 weeks (*n* = 6 per group). All values are presented as means ± S.E.M. *p < 0.05, **p < 0.01, ***p < 0.001 (HFD vs. HFD+AX) or (NC vs. NC+AX). Statistical analysis was performed using Student's *t*‐test (A‐E).

To understand the mechanism how AX improved insulin resistance in the skeletal muscle, we examined whether AX reduced oxidative stress in the plasma, skeletal muscle, liver, or adipose tissue, by TBARS assay. We found that AX reduced oxidative stress only in the epididymal white adipose tissue (eWAT), while having no such effect in the plasma, skeletal muscle, or liver (Figure 3A). Then, we examined whether AX treatment affected the respiratory quotient (RQ), which reflects the relative use of carbohydrates versus lipids as a source of energy. AX treatment significantly lowered the RQ in HFD mice compared with the non‐AX‐HFD control mice, including the all‐day, daytime, and nighttime values, showing that AX can shift the fuel preference towards fatty acids (Figure 3B). While AX did not change RQ in NC mice compared with non‐AX‐treated NC control mice (Figure 3B). This was also supported by the results of our *in vitro* study, which showed significantly increased exogenous FFA utilization in C2C12 cells following treatment with 5 μM of AX (analyzed using a seahorse flux analyzer) (Figures 3C and S4A and S4B). Consistent with these findings, we observed that AX treatment increased mitochondrial DNA copy number (Figures 3D and 3E) and protein expressions in the ETC pathway (Figures 3F and 3G and S5A and S5B) in NC and HFD mice compared with the non‐AX‐treated NC and HFD control mice, suggesting that AX treatment stimulated mitochondrial biogenesis.

**Figure 4:**
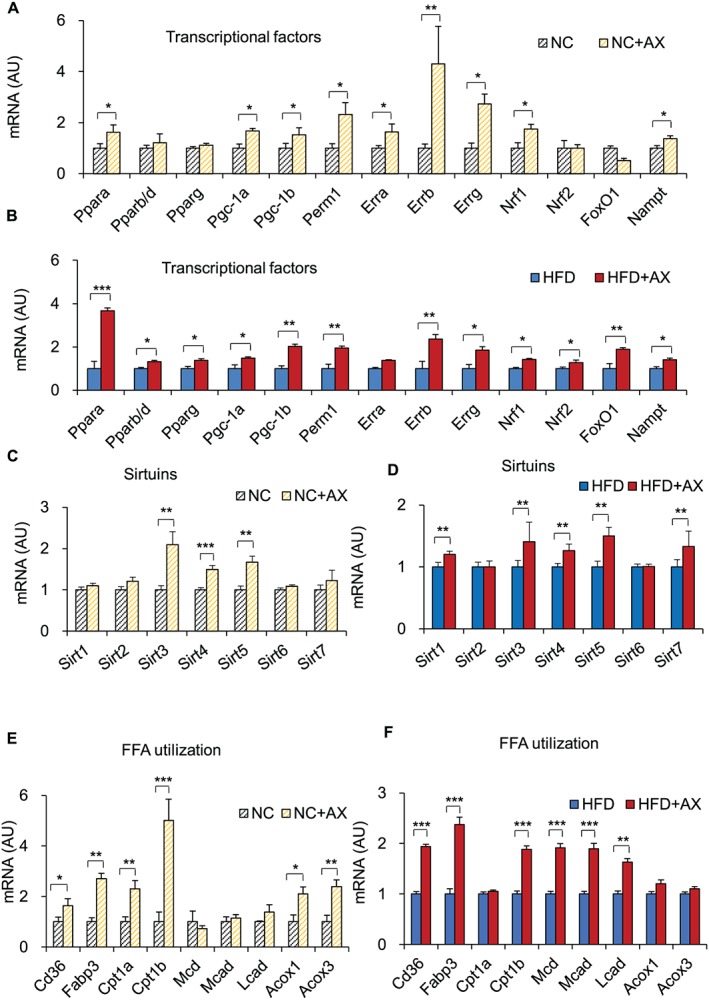
AX improved fatty acid utilization in the skeletal muscle through activation of mitochondrial function. Gene expressions in the gastrocnemius skeletal muscle relative to that of β‐actin expression, including of transcription factors related to mitochondrial energy metabolism‐related transcription factors in AX‐treated NC mice compared with the control NC mice (A) and AX‐treated HFD mice compared with control HFD mice (B), Sirtuin genes of the gastrocnemius muscle in AX‐treated NC mice compared with the control NC mice (C) and AX‐treated HFD mice compared with control HFD mice (D), FFA transport/β‐oxidation in AX‐treated NC mice compared with the control NC mice (E) and AX‐treated HFD mice compared with control HFD mice (F) (*n* = 6 per group). All values are presented as the means ± S.E.M.*p < 0.05, **p < 0.01, ***p < 0.001 (HFD vs. HFD+AX) or (NC vs. NC+AX). Statistical analysis was performed using Student's *t*‐test.

**Figure 5:**
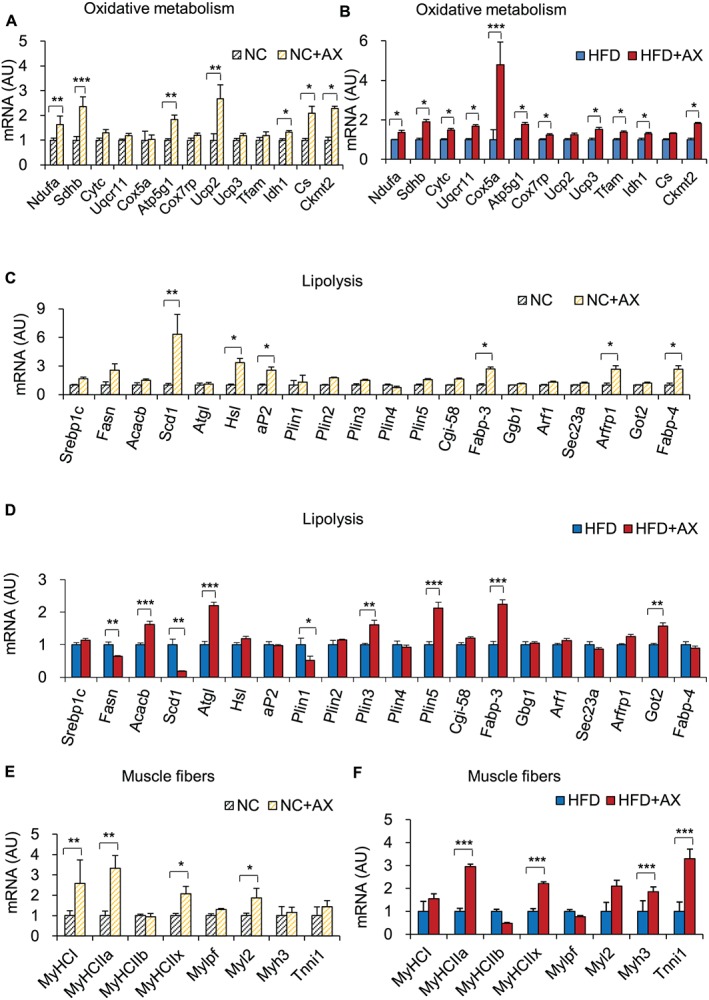
Gene expression analysis in the gastrocnemius skeletal muscle including of TCA/mitochondria function in the muscle of AX‐treated NC mice compared with the control NC mice (A) and AX‐treated HFD mice compared with control HFD mice (B), lipolysis‐related genes in the AX‐treated NC mice compared with the control NC mice (C) and AX‐treated HFD mice compared with control HFD mice (D) and skeletal muscle phenotype in the AX‐treated NC mice compared with the control NC mice (E) and AX‐treated HFD mice compared with control HFD mice (F) (*n* = 6 per group). All values are presented as the means ± S.E.M.*p < 0.05, **p < 0.01, ***p < 0.001 (HFD vs. HFD+AX) or (NC vs. NC+AX). Statistical analysis was performed using Student's *t* test.

One of the most striking results of our gene expression study of the gastrocnemius skeletal muscle was the upregulated expression of the *Pgc‐1α* gene, a major regulator of mitochondrial biogenesis and oxidative phosphorylation, and other transcription factor/regulator marker genes in the AX‐treated NC mice compared with the control NC mice (Figure 4A). Consistent with this, the gene expressions of other transcription factors/transcriptional regulators, including *Pparα*, *Pparβ/δ*, *Pparγ*, *Errα*, *Errγ*, *Foxo1*, and *Nrf1*, were also upregulated in skeletal muscle of AX‐treated HFD mice compared with HFD control mice (Figure 4B). Interestingly, AX treatment induced upregulation of the gene expression of the mitochondrial sirtuins in both AX‐treated NC and HFD mice treated compared with the non‐AX‐treated NC and HFD mice, further enhancing mitochondrial biogenesis (Figures 4C and 4D). We also found that AX treatment increased the expressions of the genes involved in the ETC and fatty acid metabolism, including mitochondrial β‐oxidation and FFA transport, *Cd36* and *Fabp3*, the mitochondrial FFA transporter *Cpt‐1β*, in the AX‐treated NC and HFD mice compared with the non‐AX‐treated NC and HFD mice (Figures 4E and 4F). AX treatment increased the expressions of the genes related to lipolysis and the TCA cycle in the NC and HFD mice compared with the non‐AX‐treated NC and HFD mice (Figures 5A and 5B). PGC‐1a is reportedly expressed strongly in slow/oxidative fibers and is more resistant to fatigue.^6^, ^41^ It has also been reported that during exercise, fast fibers are converted into slow fibers.^42^, ^43^ To investigate whether AX treatment affects the muscle mass or muscle fiber composition, we examined the muscles from AX‐treated NC and HFD mice. Both the NC and HFD mice showed significant upregulation of the genes for slow‐twitch fibers, including *MyHCΙΙa*, *MyHCIIx*, and *Myl2*, with downregulation of the fast‐twitch fiber genes in the gastrocnemius muscle after AX treatment, as compared with the control non‐AX‐treated mice (Figures 5E and 5F), suggesting that AX treatment played a pivotal role in the conversion of fast fibers to slow fibers that show improved muscle energy metabolism. Consistent with this finding, increased expressions of the endothelial marker genes, such as *Cd31* and *VE‐cadherin*, were also observed. In addition, the expression of the genes for angiogenic factors and their receptors, including *Vegfa*, *Vegfb*, *Flt1*, and *Kdr*, were also enhanced in the AX‐treated NC and HFD mice compared with the non‐AX‐treated NC and HFD mice (Figures S6A and S6B), indicating that AX remodeled myofibers into more densely vascularized ones. We also observed that the expressions of genes encoding myokines were upregulated in the skeletal muscles of AX‐treated NC and HFD mice compared with the non‐AX‐treated NC and HFD mice (Figures S6C and S6D), showing that AX treatment strengthened the muscle or increased the muscle mass.

It is known that the pyruvate dehydrogenase complex (PDC) is responsible for maintaining the metabolic flexibility in mammals.^44^ Suppression of PDC activity by pyruvate dehydrogenase kinase (PDK) is critical to the maintenance of lipid and glucose metabolism and energy homeostasis in the fed state. We examined whether AX treatment affected the activity of PDC or not. Interestingly, we also found reduced expression of *Pfkfb3* and increased expression of *Pdk4* mRNA in the AX‐treated NC and HFD mice compared with the non‐AX‐treated NC and HFD mice (Figures S7A and S7B), which reflect favored utilization of fatty acids over glucose via lowered utilization of pyruvate through inhibition of the PDC.

**Figure 6:**
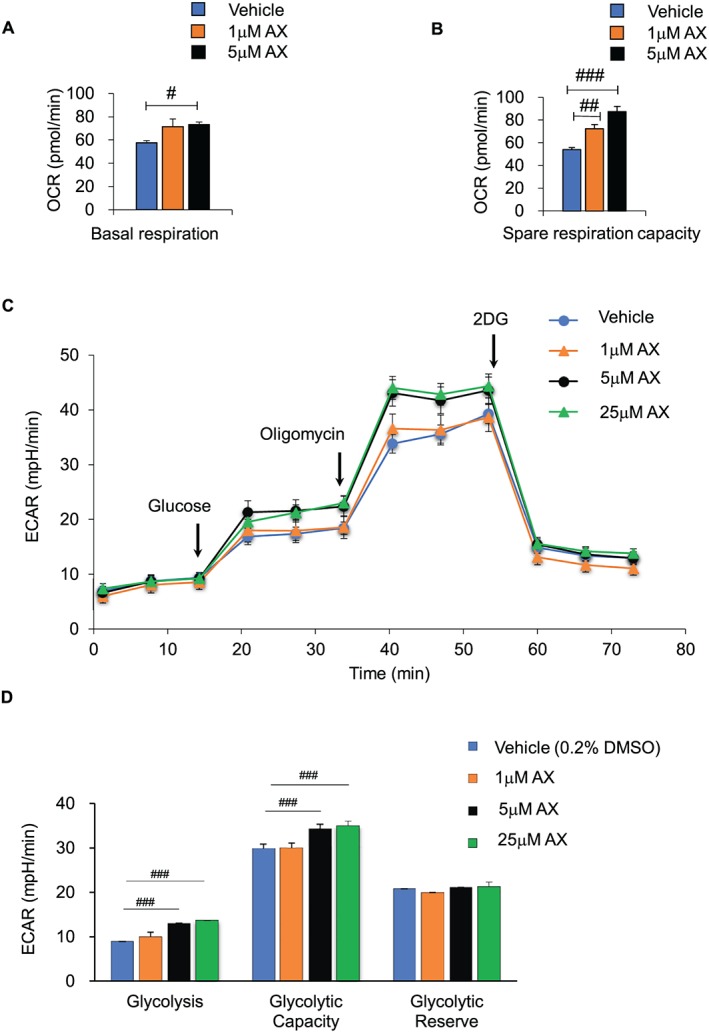
AX enhanced the mitochondrial oxidative respiration and the glycolysis capacity in the C2C12 cells. (A,B) AX enhanced the basal mitochondrial respiration rate and maximal respiratory capacity in C2C12 cells treated with vehicle, or 1 or 5 μM AX, for 24 h, using oxygen consumption rate (OCR) as a readout (*n* = 6–8 in each treatment)). (C,D) AX also enhanced the glycolysis capacity in the C2C12 cells. This parameter was evaluated with the XF Mito Fuel Flex Test kit (Agilent/Seahorse) using the XFe96 Extracellular Flux Analyzer (*n* = 11 in each treatment). All values are presented as the means ± S.E.M. #*p* < 0.05, ##*p* < 0.01, ###*p* < 0.001 (vehicle vs. AX treatment). Statistical tests one‐way ANOVA/Dunnett's test was performed.

Next, we investigated the changes in the mitochondrial functions in AX‐treated C2C12 cells, using the Seahorse flux analyzer (Figures 6A–6D and S8A– S8C). The findings revealed increased basal cellular and spare respiration in the AX‐treated cells (Figures 6A and 6B and S8). We also observed an increase in the spare respiration capacity, namely, the difference between the maximal respiration and basal cellular respiration with/without FFA (Figures 6B and S8). AX had no effect on the ATP production or proton leakage. A glycolytic stress test was performed to measure the extracellular acidification ratio (ECAR) of the C2C12 cells. We found that ECAR increased in a dose‐dependent manner in the AX‐ treated C2C12 cells (Figures 6C and 6D). These data indicated that the amelioration of insulin resistance by AX in the skeletal muscle was largely independent of its antioxidant activity. Similar to *in vivo* data, the results of *in vitro* study also supported our hypothesis that AX treatment facilitates metabolic adaptations in the skeletal muscle under the fed state.

### 3.3 AX upregulated *Pgc‐1a* expression in the skeletal muscle via AMPK activation

**Figure 7:**
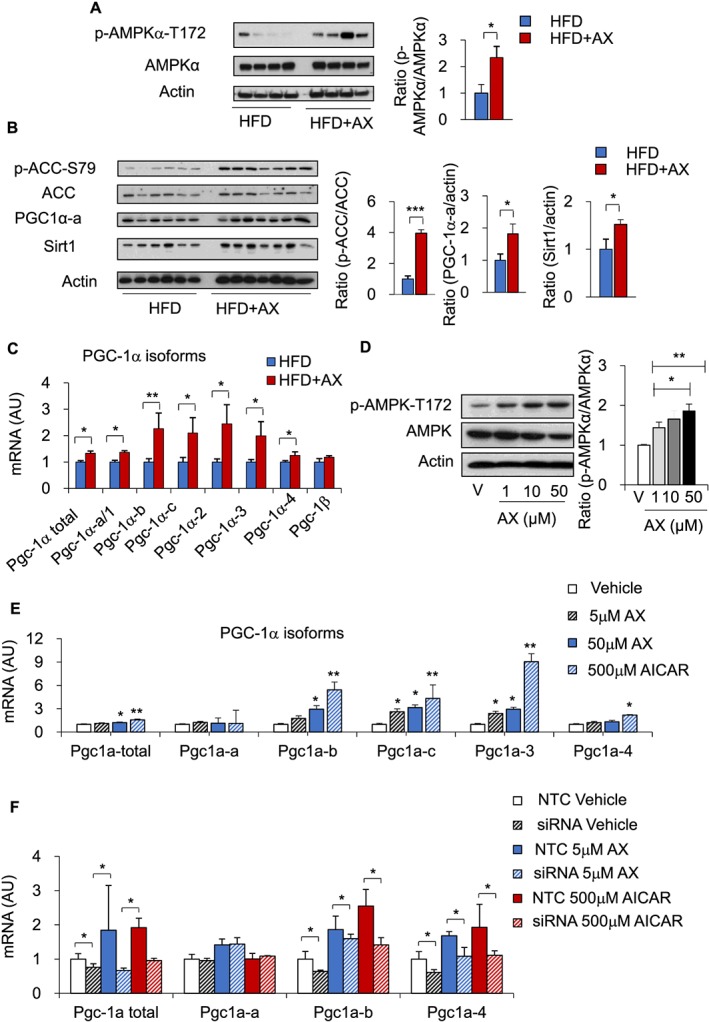
AX activated the mitochondrial metabolic pathway through AMPK activation in the mice fed HFD or HFD+AX for 24 weeks. (A,B) Western blot analysis (left) and quantification of AMPK phosphorylation (right) (A), ACC phosphorylation, PGC‐1α and Sirt1 (B) in the gastrocnemius muscle of mice fed HFD or HFD+AX (left) and quantification (right) (*n* = 6–7 per group). (C) AX induced mRNA expressions of PGC‐1α variants downstream of AMPK (HFD or HFD+AX for 24 weeks) (n = 6 per group). (D) Dose‐dependent induction of AMPK phosphorylation by AX was observed in the cultured C2C12 cells. The cells were treated with various doses of AX for up to 48h (left) and ratio of p‐AMPKα/AMPKα is given in right panel. (E) Gene expression analysis of the PGC‐1α variants downstream of AMPK treated with different doses of AX compared with vehicle control and AICAR (*n* = 3–6 in each treatment). (F) Gene expression analysis of the PGC‐1α isforms in siRNA‐AMPKα1/2 treated C2C12 cells compared with non‐target control (NTC) siRNA (*n* = 3–4 in each treatment). All values are presented as means ± S.E.M.*p < 0.05, **p < 0.01, ***p < 0.001 (HFD vs. HFD+AX) (vehicle vs. AX treatment). Statistical tests were performed as follows: (A–C,F) Student's *t* test, (D,E) one‐way ANOVA/Dunnett's test.

We next examined the mechanisms underlying the upregulation of the *Pgc‐1α* gene and other genes controlling mitochondrial biogenesis in the AX‐treated HFD mice. AMPK is known to induce *Pgc‐1α* expression and to directly enhance its activity through phosphorylation. It has also been reported to activate Sirt1 through Nampt‐mediated increase of NAD^+^, thereby activating PGC‐1α.^45^ In fact, we observed that AX stimulated both *Nampt* and *Sirt1* gene expressions (Figure 4). Thus, we examined the AMPK activity in the gastrocnemius muscle of the AX‐treated HFD mice and found increased phosphorylation of AMPKα and its downstream target, ACC, in the muscle (Figures 7A and 7B). The amount of PGC‐1 protein was also increased in the AX‐treated HFD mice (Figure 7B). AX treatment induced the expressions of the *Pgc‐1α‐b*, *Pgc‐1α‐c*, *Pgc‐1α‐2*, and *Pgc‐1α‐3* genes that lie downstream of AMPK in the AX‐treated NC and HFD mice compared with the non‐AX‐treated NC and HFD mice (Figures 7C and S9). Thus, AX treatment augmented the expressions of the genes lying downstream of AMPK in both the HFD and NC mice.

We next examined whether AX treatment of C2C12 cells activated AMPK. AX treatment for 48 h increased the phosphorylation of AMPK in a dose‐dependent manner (Figure 7D). Consistent with this finding, *in vitro* AX‐treated C2C12 showed dose‐dependent upregulated expression of *Pgc‐1α‐b*, *Pgc‐1α‐c*, *Pgc‐1α‐2*, and *Pgc‐1α‐3*, which lie downstream of AMPK, compared with the vehicle (Figure 7E). In order to validate our hypothesis, we knocked down the AMPK in the C2C12 cells by using AMPKa1/2 siRNA. We assessed the effect of AX and AICAR in genetically depleted C2C12 cells (siRNA‐mediated knockdown of AMPK in C2C12 cells). First, we confirmed siRNA‐mediated knock down of AMPK by gene expression analysis (Figure S10A). The effect of AX and AICAR on mitochondrial biogenesis was lost in genetically AMPK depleted C2C12 cells (Figure 7F). We also showed that the effect of AX on mitochondrial biogenesis was lost in genetically AMPK‐depleted C2C12 cells (Figure S10B), suggesting that AX enhanced mitochondrial biogenesis and muscle adaptation via activation of AMPK pathway. These data support the hypothesis that AX directly stimulates AMPK activity, thereby inducing *Pgc‐1α* gene expression and stimulating PGC‐1α activity. Increased PGC‐1α activity leads to enhanced mitochondrial biogenesis and oxidative phosphorylation in the skeletal muscle, resulting in amelioration of insulin resistance in the muscle.

### 3.4 AX augmented the effect of exercise training on glucose intolerance in the HFD mice

AX is a strong antioxidant and could scavenge physiological reactive oxygen species (ROS) generated during exercise training, thereby canceling the beneficial effects of exercise with regard to endurance and glucose tolerance. Previous reports have also suggested that an exercise tolerance test is important to assess the metabolic adaptations in muscle and exercise‐induced ROS generation have beneficial effect on metabolic adaptation in muscle.^32^, ^46^, ^47^, ^48^ Thus, we next examined whether AX affected the training‐induced increase in exercise tolerance. Because AX ameliorated insulin resistance in HFD mice, the skeletal muscle function in the mice was examined using a single‐bout of endurance exercise tolerance test. Accordingly, we compared the results of the exercise tolerance test in the NC and HFD mice treated with AX compared with non‐AX‐treated NC and HFD mice. We found that AX‐treatment increased the endurance of the both NC and HFD mice compared with non‐AX‐treated NC and HFD mice (Figures S11A and S11B). Interestingly, AX‐treated HFD mice that were subjected to daily exercise using a treadmill and wheel ran for a longer distance than the non‐AX‐treated control HFD mice (Figure S11C), showing that AX administration further enhanced the endurance of these mice. We also observed that the AX‐treated HFD mice showed improved the glucose tolerance after regular daily training (Figure S11D and S11E). These data indicated that AX treatment did not compromise the effect of daily training on the endurance but rather improved exercise‐induced glucose tolerance and endurance.

Hydrogen peroxide is known to play an important role in exercise‐induced remodeling of the skeletal muscle.^49^, ^50^ Thus, we next examined whether AX affected H_2_O_2_ signaling in the C2C12 cells. Our study revealed that AX had no effect on H_2_O_2_‐induced AMPK phosphorylation (Figure S12A); this finding was consistent with the absence of interference by AX with the effect of daily exercise on the endurance.

## 4 Discussion

We demonstrated that AX activated AMPK in the muscle and upregulated the expressions of a transcriptional coactivator and transcriptional factors, thereby inducing mitochondrial remodeling, including increased mitochondrial oxidative phosphorylation and FFA metabolism. Furthermore, AX treatment increased the endurance and attenuated glucose intolerance in HFD mice. Thus, we identified AX as a potent exercise mimetic. Both *in vivo* and *in vitro* studies revealed that AX administration enhanced AMPK activation in the skeletal muscle, along with upregulation of the expressions of the genes associated with metabolic and mitochondrial oxy‐phosphorylation, independently of its antioxidant activities. It has been reported that overexpression of *Ppar‐δ* in the muscle induces expression of the mitochondrial gatekeeper proteins such as *Cpt‐1β* and *Pdk4,* which regulate FA metabolism in the muscle and promote running endurance.^9^, ^10^, ^51^, ^52^, ^53^ Consistent with these reports, our study showed that AX treatment upregulated the expressions of *Ppar‐δ* and *Pdk4* in the muscle, which induced mitochondrial gatekeeper proteins to stimulate mitochondrial biogenesis, and also increased the running time and endurance, thereby further ameliorating insulin resistance in the HFD mice. The issue of whether antioxidant treatment could attenuate or eliminate exercise‐induced adaptive beneficial effects in the skeletal muscle,^54^, ^55^ called the “hormesis” effect, 56 is under debate. Despite its potent antioxidant activity, AX reportedly enhanced exercise performance and recovery from fatigue and prevention of damage associated with high‐intensity exercise. AX administration further enhanced the beneficial effects of exercise endurance. This effect may be attributable to the effect of AX of stimulating mitochondrial biogenesis through activation of the AMPK‐PGC‐1α pathway. It also appears that AX does not scavenge hydrophilic ROS, such as O_2_‐ and H_2_O_2_, produced during exercise.^57^, ^58^ Interestingly, AX did not interfere with H_2_O_2_‐induced AMPK activation either. AX did not appear to affect the signals initiated by muscle contraction‐associated ROS, which contribute to exercise‐induced beneficial remodeling of the muscle. On the other hand, AX may exert a protective effect through its potent antioxidant activity when the intensity of exercise is high and a large amount of ROS is generated. AX is also known as a strong antioxidant against singlet oxygen and lipid peroxidation,^59^, ^60^ and to reduce muscle oxidative damage and the inflammation associated with it after severe endurance running 40 or young soccer players.^61^ It is possible that AX somehow sequesters ROS that cause damage, but not the ROS that induce beneficial effects. Previous reports suggested that exercise‐induced generation of ROS not only causes oxidative damage, but that the ROS also function as signaling molecules to facilitate beneficial molecular adaptations.^32^, ^48^ To rule out the possibility that AX might mitigate the ROS‐mediated positive effects of exercise training by activating several molecules, including AMPK and PGC‐1α, in the muscle, we examined whether AX had any effect on H_2_O_2_‐induced AMPK phosphorylation. Then, we found that AX did not affect H_2_O_2_‐induced AMPK activation. We have shown that AX does not attenuate oxidative stress represented by TBARS by HFD in skeletal muscle *in situ* but attenuated TBRAS in the eWAT of HFD mice (Figure 3A). Interestingly, we found reduced expression of inflammatory marker genes in the eWAT of AX‐treated HFD mice (Figure S12B), while the expression of inflammatory marker genes remain unchanged in the skeletal muscle (Figure S12C), showing that AX playing antiinflammatory role in adipose tissue.

How does AX activate AMPK in the skeletal muscle? According to previous reports natural compounds, endogenous hormones and synthetic AMPK activators can be divided into four major classes according to their mechanisms of action: (1) indirect activators, such as including resveratrol and quercetin, that inhibit cellular ATP production and increase the cellular AMP:ATP and ADP:ATP ratios;^39^, ^62^, ^63^, ^64^ (2) compounds, such as AICAR, that are prodrugs converted into AMP analogs; (3) endogenous hormones, such as adiponectin, that activate AMPK through specific receptors on the plasma membrane of the target cells;^65^, ^66^, ^67^ and (4) direct AMPK activators, such as thienopyridone A769662, which bind to the β subunit carbohydrate‐binding module called ADaM, to directly activate AMPK, mimicking the effects of AMP to cause allosteric activation, and protect against Thr172 dephosphorylation.^68^ It remains unknown whether AX activates AMPK by one of these four major mechanisms, or by some other novel mechanism. We demonstrated that AX treatment significantly reduced oxidative stress and adipose tissue inflammation, which may explain, at least in part, the effect of AX of ameliorating insulin resistance in the HFD mice. AX shifted the macrophages polarity toward M2‐like macrophages by reducing oxidative stress in the adipose tissue.^37^, ^38^, ^69^, ^70^, ^71^ It is possible that AX prevents lipid peroxidation within lipid droplets in adipocytes via its antioxidant activity, thereby reducing adipocyte death and consequently decreasing the number of infiltrating macrophages. Furthermore, it is considered that AX suppresses inflammatory signaling, such as that via the NFκB pathway, in macrophages.^28^, ^72^ Hypertrophic adipocytes and infiltrating macrophages produce excessive amounts of O_2_‐ and H_2_O_2_, thereby generating highly toxic ROS, such as hydroxyl radicals and/or free radicals from lipid radical chain reaction.^73^ AX can scavenge these kinds of ROS in the adipose tissue, which could explain why AX scavenges ROS selectively in the adipose tissue of obese mice. Interestingly, a recent study suggests that production of ROS may be necessary for promoting an extended lifespan;^74^ this study illustrates that ROS production with reduced ubiquinone, and possibly through respiratory reverse electron transport toward complex I, can extend the lifespan of *Drosophila*.^74^ AX has also been reported to have the ability to extend the lifespan of wild‐type *C.elegans* in a dose‐dependent manner,^75^ although it remains unclear whether this lifespan extension is due to the remodeling of mitochondria. These mechanisms were largely similar to those of known AMPK activators such as biguanides, adiponectin, and resveratrol.^39^, ^62^, ^65^, ^66^ These compounds also improved insulin resistance by inducing *Pgc‐1α* gene expression, which is a master regulator of mitochondria‐related genes, via activation of the AMPK/Sirt1 pathway.^63^, ^65^ Pharmacological approaches for type 2 diabetes or antiaging targeting this pathway have been attempted recently.^64^, ^67^ However, it is difficult to exclude the possibility that AX potentially exerts its effects via entirely different mechanisms from these compounds. In addition to its antioxidant activity, it has been shown that AX also acts as a weak partial modulator of PPARγ *in vitro*.^76^ In fact, in this study also, HFD mice treated with AX showed partially increased expressions of *Pparγ,* suggesting an insulin‐sensitizing effect of AX in the HFD mice.

In summary, this study revealed that AX treatment (i) significantly ameliorated insulin resistance and glucose intolerance through regulation of AMPK activation in the muscle, independent of its antioxidant activity, (ii) stimulated mitochondrial biogenesis in the muscle, (iii) enhanced endurance and running time and exercise‐induced FA metabolism and muscle remodeling, and (iv) exerted antiinflammatory effects via its antioxidant activity in the adipose tissue. AX could be a highly promising natural compound with therapeutic potential to prevent the development of type 2 diabetes and obesity‐related disorders, based on the findings in rodents.

## Conflict of Interest

None declared.

## Authors' contributions

Y. N. and A. N. performed the experiments, acquired and analyzed the data, and wrote the manuscript. Y. I, T. K, S. Y, and A.T. helped in genotyping and RT‐PCR analysis. Y. K, I. U, and S.F. helped to acquire and analyze the data. Y. O and T. W helped in RQ analysis. T. S, M. S, and J. I. helped in discussing the manuscript. K. T. helped in hyperisnulinemic‐euglycemic study. K. Tobe supervised the project. All the authors approved the final version of the paper for publication.

## Additional Information

Competing financial and nonfinancial interests: Y.N. is currently employed by Fuji Chemical Industries Co, Ltd. All other authors declare that there is no duality of interest associated with this manuscript.

## Ethical standard

All institutional and national guidelines for the care and use of laboratory animals were followed.

## Supporting information

Table S1: SYBR Green primers sequence

Data S1 Supporting Information
