# Supplementary material for: Astaxanthin stimulates mitochondrial biogenesis in insulin resistant muscle via activation of AMPK pathway
Source: J Cachexia Sarcopenia Muscle. 2020 Jan 31;11(1):241–58. doi: 10.1002/jcsm.12530 (PMC7015247; doi:10.1002/jcsm.12530)
Supplement: Supplementary file 1 — Table S1: SYBR Green primers sequence [file JCSM-11-241-s001.pdf]

Table S1; SYBR Green primers sequence

Gene Name	Fw. Seq.	Rv. Seq.
Abhd5 (Cgi-58)	GACACCAGGTAGGAAGCCAC	GCTTCGAGGTGTGTCCCTG
Acacb	TGTTCTCGGCCTCTCTTCAC	GAGGCTGCATTGAACACAAG
Acox1	GGAATTTGGCATCGCAGACC	ACATGCCCAAGTGAAGGTCC
Acox3	GCTCGGTAGGCACTAAGAGG	CTTCTGAGAAACGGGGACAA
AMPKα1(Prkaa1)	ACCAGGTCATCAGTACACCA	TCCTTTTCGTCCAACCTTCCA
AMPKα2 (Prkaa2)	GGCAAAGTGAAGACTACCAGG	CTTCAACCCGCCCATGTTTG
Angptl4	GCTCATTGGCTTGACTCCCA	GCCTCCATCTGAAGTCATCTCA
aP2	GATGCCTTTGTGGGAACCTG	GAATTCCACGCCCAGTTTGA
Arf1	GGAGGTCTTTGGCCAGTATCG	ACATTGAAACCAATGGTGGGA
Arfrp1	GGATGTACACGCTGCTTTCG	GTGCCAATGTTTAGACCCACG
Atrogin-1(Fbxo32)	CTCAGGGATGTGAGCTGTGA	GGGGGAAGCTTTCAACAGAC
β-actin	GCCGGGACCTGACAGACTAC	AACCGCTCGTTGCCAATAGT
Bdnf	GACGTTTACTTCTTTCATGGGC	TGAGTCTCCAGGACAGCAAA
Bmp1	TAGTCATACCAGCACAGGCG	CTACTCTGCCCACATGCACT
Cox7rp	TTTGGTTGGTGTGGCAAATA	AGTTTCACGCAGAAGTTGGC
Cpt1a	TTGGAAGTCTCCCTCCTTCA	GCCCATGTTGTACAGCTTCC
Cs	AAGGACGAGGCAGGATGAG	TGCAGCTGTAGCTCTCTCCC
Epas1 (Hif2a)	CATCACGGGATTTCTCCTTC	AGGGCCACAGCAAAGAGAG
Erra	CAGCTGTACTCGATGCTCCC	AGCTCTCTACCCAAACGCCT
Errb	TGAAGGAGCCGCAACTAGAG	GCTGGAACACCTGAGGGTAA
Errg (Nr3b3)	TTGTACTTCTGCCGACCTCC	TGAGATCACAAAGCGCAGAC
Fabp-3	CCGTTCTTCTCGATGATGGT	CGGTACCTGGAAGCTAGTGG
Fabp4	AAATCACCGCAGACGACAGG	ATAACACATTCCACCACCAGC
Fabp4	AAATCACCGCAGACGACAGG	ATAACACATTCCACCACCAGC
Fam132b (Ctrp15, Myonectin)	TGCTTGGATGCTGTTCGTCAA	CAGATGGGATAAAGGGGCCTG
Flt1 (Vegfr1)	TGCTGGGTGCCTTTTAAACT	ATGGTCAGCTGCTGGGAC
Fst (Follistatin)	GACAATGCCACATACGCCAG	GTTTCTTCCGAGATGGAGTTGC
Fstl1	GCCAGCTCCACAAAACACAT	GAGCACGATGTGGAAACGAT
Gbf1	TCCAGAAATGGACGAAGGAA	CACTGGATGAAGAACGGGAT
Glut4(slc2a4)	CAGTGTTCCAGTCACTCGCT	CAGCGCCTGAGTCTTTTCTT
Got2(Fabpm)	ACTTGCGGAAGCAATGGTTG	GTGATGTGCTGCCAGTTGTG
Gpi1	AATGGAAAGTCCAATGGCTG	ACTTTGTCGCCCTGTCTACG
H6pd (G6pd)	TGCTGATGTTGAGAGGCAGT	GGAGACCATAGATGCGGAA
Idh1	CTCTGCAGCATCTTTGGTGA	TGACACGAATCATTTGGGAA
Il6ra	ACCAGGTGATCATTCAGGGA	TGGGTGTACTCTGGCTCACA
Insl6	GATTCACAAACCCAGTCCCTG	CCTTAGGAAGCAACTCCGTCT
Kdr (Vegfr2)	TCCAGAATCCTCTTCCATGC	CCAGAGACCCTCGTTTTCAG
Lif	GACCACGGCTCCAGTATATAAATC	GCAGCAGTAAGGGCACAATC
Mmp2	CAGGGCACCTCCTACAACAG	CAGTGGACATAGCGGTCTCG
Mstn	GAGCCCAGGCACTGGTATTT	AGGGATTCAGCCCATCTTCTC
MtDNA1	CCGCAAGGGAAAGATGAAAGAC	TCGTTTGGTTTCGGGGTTTC
Myh3	GACTCAGCCGACACCATGAG	CTCTGGCTTAACCACCAGGG
MyHCI (Myh7 Slow)	CTCAGGTGGCTCCGAGAAAG	TGGCTGAGCCTTGGATTCTC
MyHCIIa (Myh2)	GCAAGAAGCAGATCCAGAAAC	GGTCTTCTTCTGTCTGGTAAGTAAGC
MyHCIIb (Myh4 Fast)	TCTGGTAACACAAGAGGTGC	AAAAGGCTTGTTCTGGGCCT
MyHCIIx(Myh1 Mod)	GCAACAGGAGATTTCTGACCTCAC	CCAGAGATGCCTCTGCTTC
Mylpf	GTATGTTAAGGGCTGCCGGG	TTACAGTGAACGCCTCCTTGA
MyoG	GTGAATGCAACTCCCACAGC	CGCGAGCAAATGATCTCCTG
Nlrp3	GGACCAGCCAGAGTGGAATGA	AACCTGCTTCTCACATGTCGT
Nos3 (eNOS)	GTTGTACGGGCCTGACATTT	GGTCCTGTGCATGGATGAG
Pck2	TGTCACCATAACCTCGTGGC	GTGGTACCCCTTTGGGTCG
PCK2	TGTCACCATAACCTCGTGGC	GTGGTACCCCTTTGGGTCG
Pfkfb3	CCACCATCACAATCACGGT	ACAGCTACGAAGATGCCGTT
Pfkm	GCCCGTGAAGATACCAACTC	CCATGAAGAGCATCATGCAG
Pgc1-1a	AGCCGTGACCACTGACAACGAG	GCTGCATGGTTCTGAGTGCTAAG
Pgc-1a total_Sp	TGATGTGAATGACTTGGATACAGACA	GCTCATTGTTGTACTGGTTGGATATG
Pgc1a-1	GGACATGTGCAGCCAGACTCT	CACTTCAATCCACCCAGAAAGCT
Pgc1a-2	CCACCAGAATGAGTGACATGGA	GTTCAGCAAGATCTGGGCAAA
Pgc1a-3	AAGTGAGTAACCGGAGGCATTC	TTCAGGAAGATCTGGGCAAAGA
Pgc1a-4	TCACACCAAACCCACAGAAA	CTGGAAGATATGGCACAT
Pgc1a-a	GGGACATGTGCAGCCAAGA	AAGAGGCTGGTCCTCACCAA
Pgc1a-b	GACATGGATGTTGGGATTGTCA	ACCAACCAGAGCAGCACATTT
Pgc1a-c	TGAGTAACCGGAGGCATTCTCT	TGAGGACCGCTAGCAAGTTTG
Pgc-1β	TCCTGTAAAAGCCCGGAGTAT	GCTCTGGTAGGGGCAGTGA
Pkm2	TCGCATGCAGCACCTGATT	CCTCGAATAGCTGCAAGTGGTA
Plin1	GGTGTTACAGCGTGGAGAGTAA	TCTGGAAGCACTCACAGGTC
Plin2 (Adfp)	CAGCTCTCCTGTTAGGCGT	CGGAGGACACAAGGTCGTAG
Plin3	TACTTTGTCCGGTTGGGCTC	GATTCCATCAGGCCGAGCA
Plin4	GAGGGACCCTTTACCCTGAC	CTCAAGGTCTTGCCTTTGGAT
Plin5 (Oxpat)	CCATCAGACATGGTGGTGACA	GCTAGCTCAGCCTCAGTCAT
Scd1	GCCGAGCCTTGTAAGTTCTG	CCTCCTGCAAGCTCTACACC
Sec23a	CACGGAGCTTCTAGACAGAAAT	ATTCCAACTGAACCGGACCC
Sirt2	GGCTCAGGATTCAGACTCGG	GCTCGTCTAGAAGACGCTCC
Sirt5	ACAGGGCGGTTAAGAAGTCC	GGCCGAGTTTAACATGGAGA
Sirt6	ACCCACAAAACATGACCGCC	TTTGTCTAGCACGCAGGGTC
Sirt7	CCGTCGACCCAAACTCTACA	CTGCCACCGGTTGTAGACAG
Sparc (Osteonectin)	CCAGGCAAAGGAGAAAGAAG	ATTCCTGCAGCCCTTCAGAC
Srebp2	GATGATCACCCCGACGTTCA	TGCAGGACTTGAAAGCTGGT
SRF	GCAGTGATGTATGCCCCCAC	CAGCCATCTGGTGAAGCTGAA
Stars (Abra)	GGCTACGGAGGGGATATGGA	CAGAGTACCTGTTTGCCTGGG
Tf2b	TGGAGATTTGTCCACCATGA	GAATTGCCAAACTCATCAAAACT
Ucp2	TGGAAAATCGAGGGGATCGG	GGAGAAACGGGGACCTTCAA
VE-cadherin	ACGGACAAGATCAGCTCCTC	TCTCTTCATCGATGTGCATT
Vegfa	AAAAACGAAAGCGCAAGAAA	TTTCTCCGCTCTGAACAAGG
Vegfb	ACACCAAGTCCGAATGCAGAT	GTATGGCAACCCTGTCTGGCT
