# Supplementary material for: Astaxanthin stimulates mitochondrial biogenesis in insulin resistant muscle via activation of AMPK pathway
Source: J Cachexia Sarcopenia Muscle. 2020 Jan 31;11(1):241–58. doi: 10.1002/jcsm.12530 (PMC7015247; doi:10.1002/jcsm.12530)
Supplement: Supplementary file 2 — Data S1 Supporting Information [file JCSM-11-241-s002.pptx]

## Slide 1
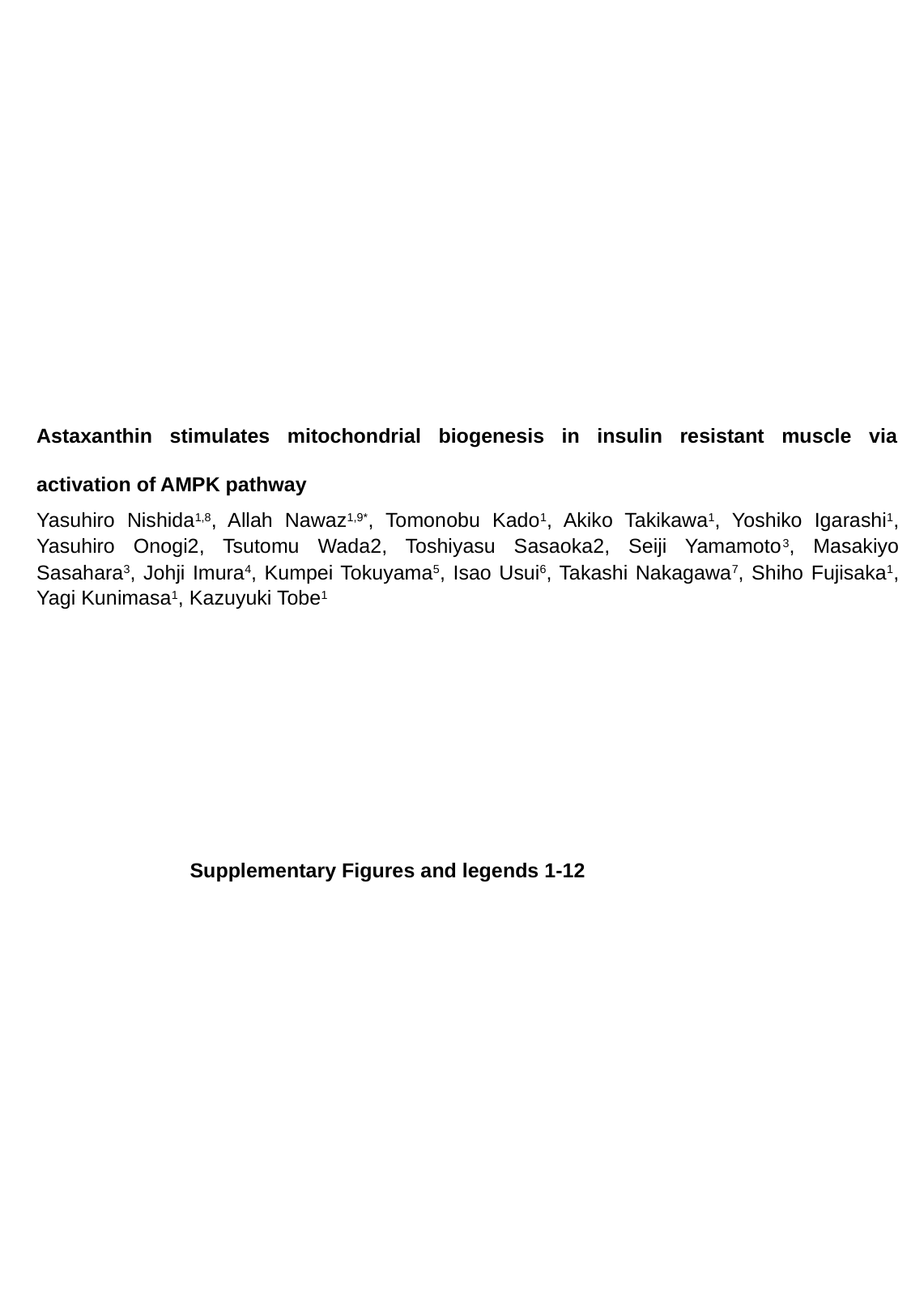

Astaxanthin stimulates mitochondrial biogenesis in insulin resistant muscle via activation of AMPK pathway
Yasuhiro Nishida1,8, Allah Nawaz1,9*, Tomonobu Kado1, Akiko Takikawa1, Yoshiko Igarashi1, Yasuhiro Onogi2, Tsutomu Wada2, Toshiyasu Sasaoka2, Seiji Yamamoto3, Masakiyo Sasahara3, Johji Imura4, Kumpei Tokuyama5, Isao Usui6, Takashi Nakagawa7, Shiho Fujisaka1, Yagi Kunimasa1, Kazuyuki Tobe1
Supplementary Figures and legends 1-12

## Slide 2
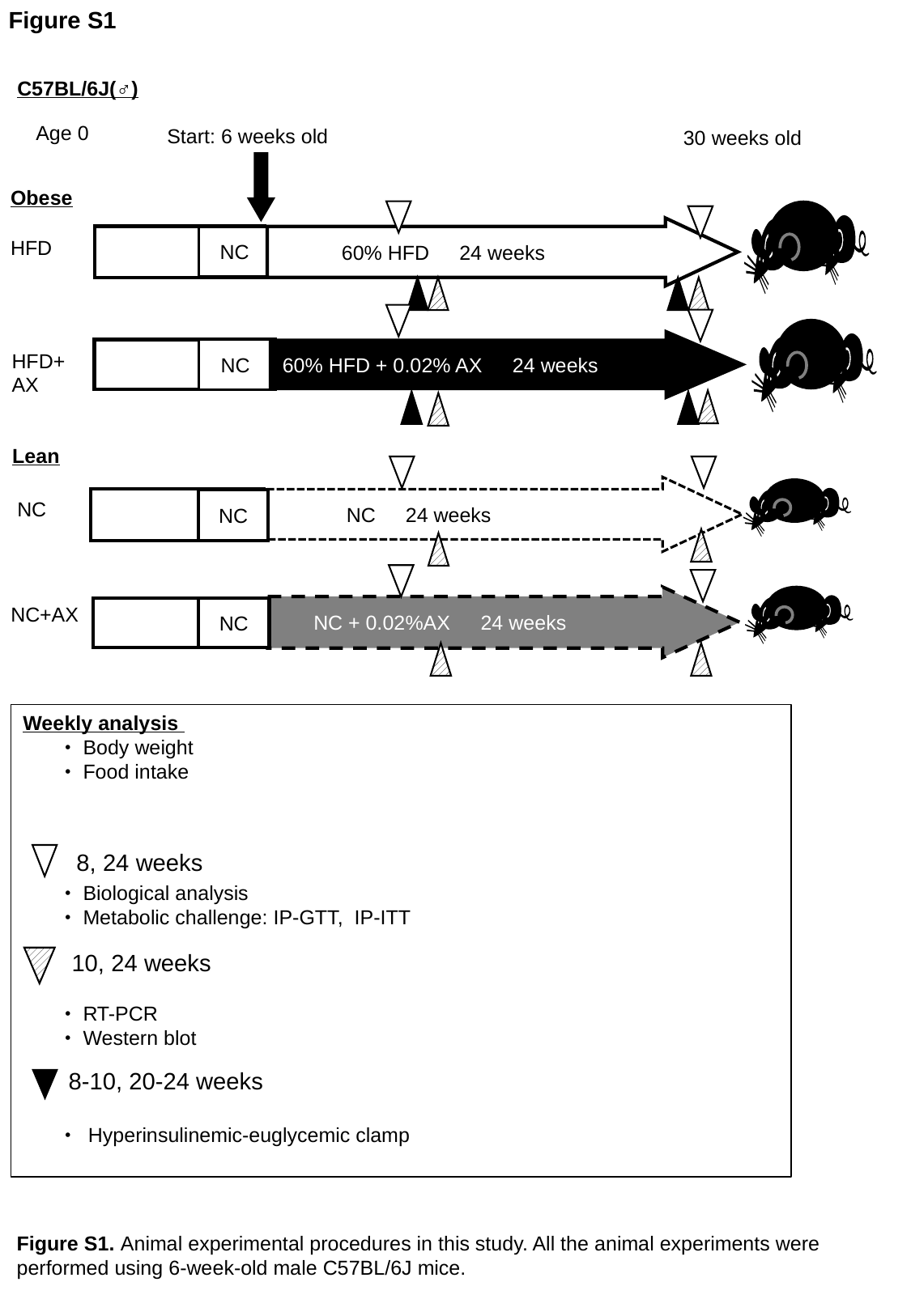

Figure S1
C57BL/6J(♂)
Age 0
Start: 6 weeks old
30 weeks old
Obese
 60% HFD　24 weeks
NC
HFD
60% HFD + 0.02% AX　24 weeks
NC
HFD+
AX
Lean
 NC　24 weeks
NC
NC
 NC + 0.02%AX　24 weeks
NC+AX
NC
Weekly analysis
・Body weight
・Food intake
・Biological analysis
・Metabolic challenge: IP-GTT, IP-ITT
・RT-PCR
・Western blot
・ Hyperinsulinemic-euglycemic clamp
8, 24 weeks
10, 24 weeks
8-10, 20-24 weeks
Figure S1. Animal experimental procedures in this study. All the animal experiments were performed using 6-week-old male C57BL/6J mice.

## Slide 3
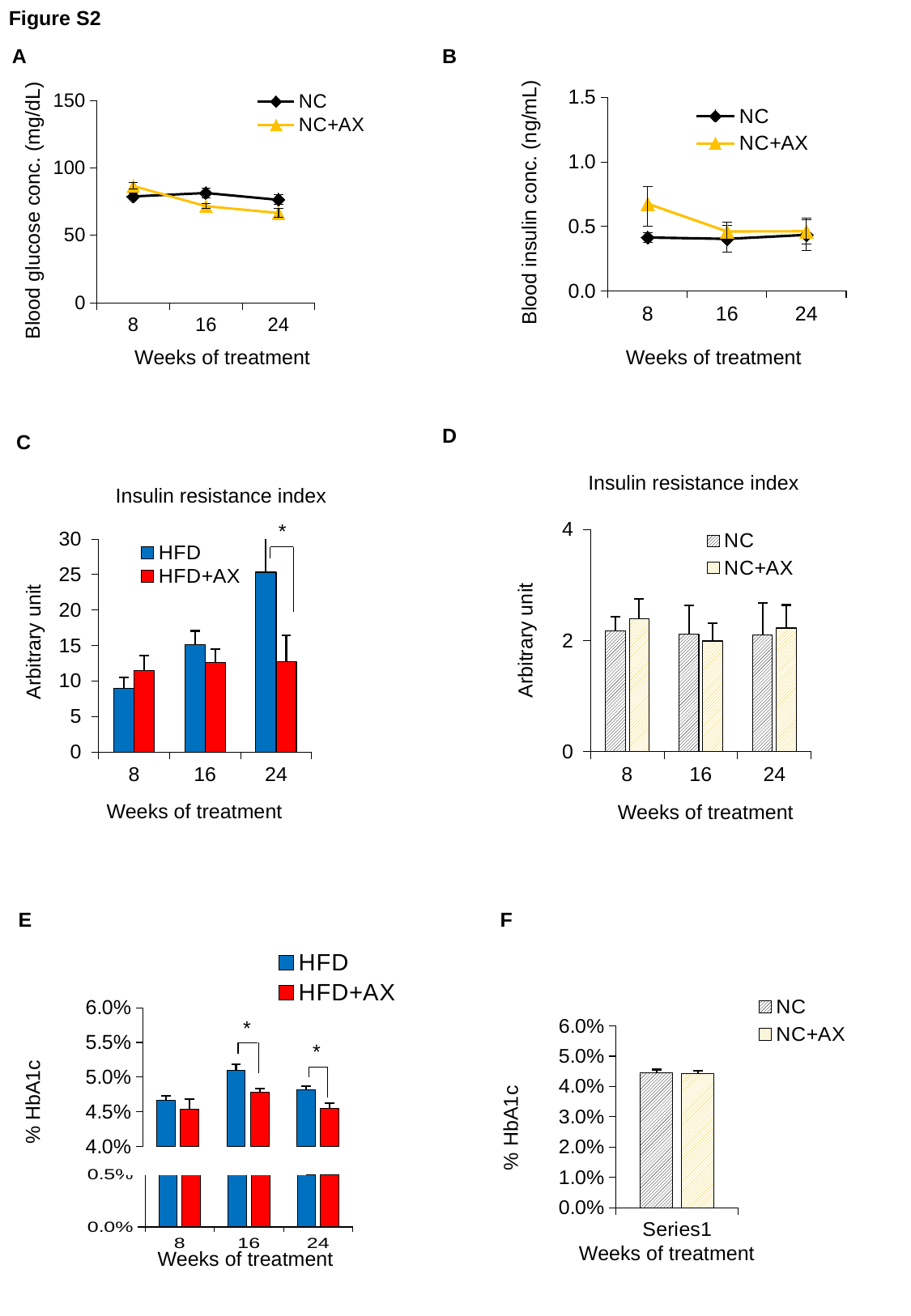

Figure S2
A
B
### Chart
| Category | NC | NC+AX |
|---|---|---|
| 8 | 0.4141329617511171 | 0.6760915524500888 |
| 16 | 0.40335854126412113 | 0.4611648906487972 |
| 24 | 0.43487282102190267 | 0.46274156170450925 |
### Chart
| Category | NC | NC+AX |
|---|---|---|
| 8 | 78.83333333333333 | 86.57142857142857 |
| 16 | 81.4 | 71.6 |
| 24 | 76.3 | 66.6 |Blood insulin conc. (ng/mL)
Blood glucose conc. (mg/dL)
Weeks of treatment
Weeks of treatment
D
C
Insulin resistance index
Insulin resistance index
*
### Chart
| Category | NC | NC+AX |
|---|---|---|
| 8 | 2.177596241722402 | 2.3884428661046435 |
| 16 | 2.1169995228630594 | 1.992746143658979 |
| 24 | 2.1054502063101004 | 2.2251390978699708 |
### Chart
| Category | HFD | HFD+AX |
|---|---|---|
| 8 | 8.961062465763773 | 11.472894305576796 |
| 16 | 15.155461798780692 | 12.610413906350633 |
| 24 | 25.33801643977417 | 12.742512175008663 |Arbitrary unit
Arbitrary unit
Weeks of treatment
Weeks of treatment
E
F
### Chart
| Category | HFD | HFD+AX |
|---|---|---|
| 8 | 0.046599999999999996 | 0.0454 |
| 16 | 0.051000000000000004 | 0.04780000000000002 |
| 24 | 0.048166666666666684 | 0.04549999999999999 |
### Chart
| Category | NC | NC+AX |
|---|---|---|
| | 0.04440000000000005 | 0.0442 |*
*
% HbA1c
% HbA1c
### Chart
| Category | HFD | HFD+AX |
|---|---|---|
| 8 | 0.046599999999999996 | 0.045399999999999996 |
| 16 | 0.051000000000000004 | 0.047799999999999995 |
| 24 | 0.04816666666666666 | 0.04549999999999999 |Weeks of treatment
Weeks of treatment

## Slide 4
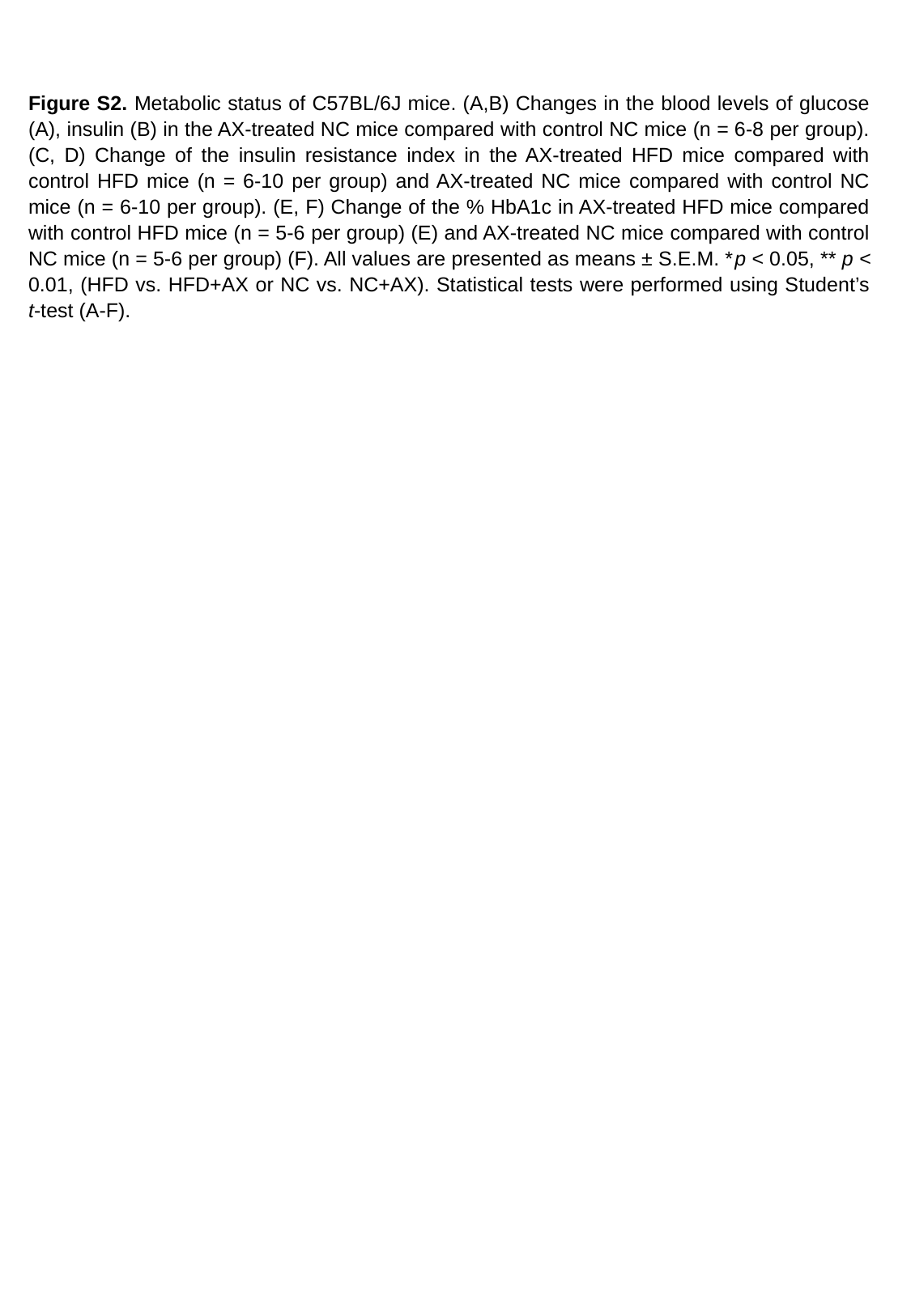

Figure S2. Metabolic status of C57BL/6J mice. (A,B) Changes in the blood levels of glucose (A), insulin (B) in the AX-treated NC mice compared with control NC mice (n = 6-8 per group). (C, D) Change of the insulin resistance index in the AX-treated HFD mice compared with control HFD mice (n = 6-10 per group) and AX-treated NC mice compared with control NC mice (n = 6-10 per group). (E, F) Change of the % HbA1c in AX-treated HFD mice compared with control HFD mice (n = 5-6 per group) (E) and AX-treated NC mice compared with control NC mice (n = 5-6 per group) (F). All values are presented as means ± S.E.M. *p < 0.05, ** p < 0.01, (HFD vs. HFD+AX or NC vs. NC+AX). Statistical tests were performed using Student’s t-test (A-F).

## Slide 5
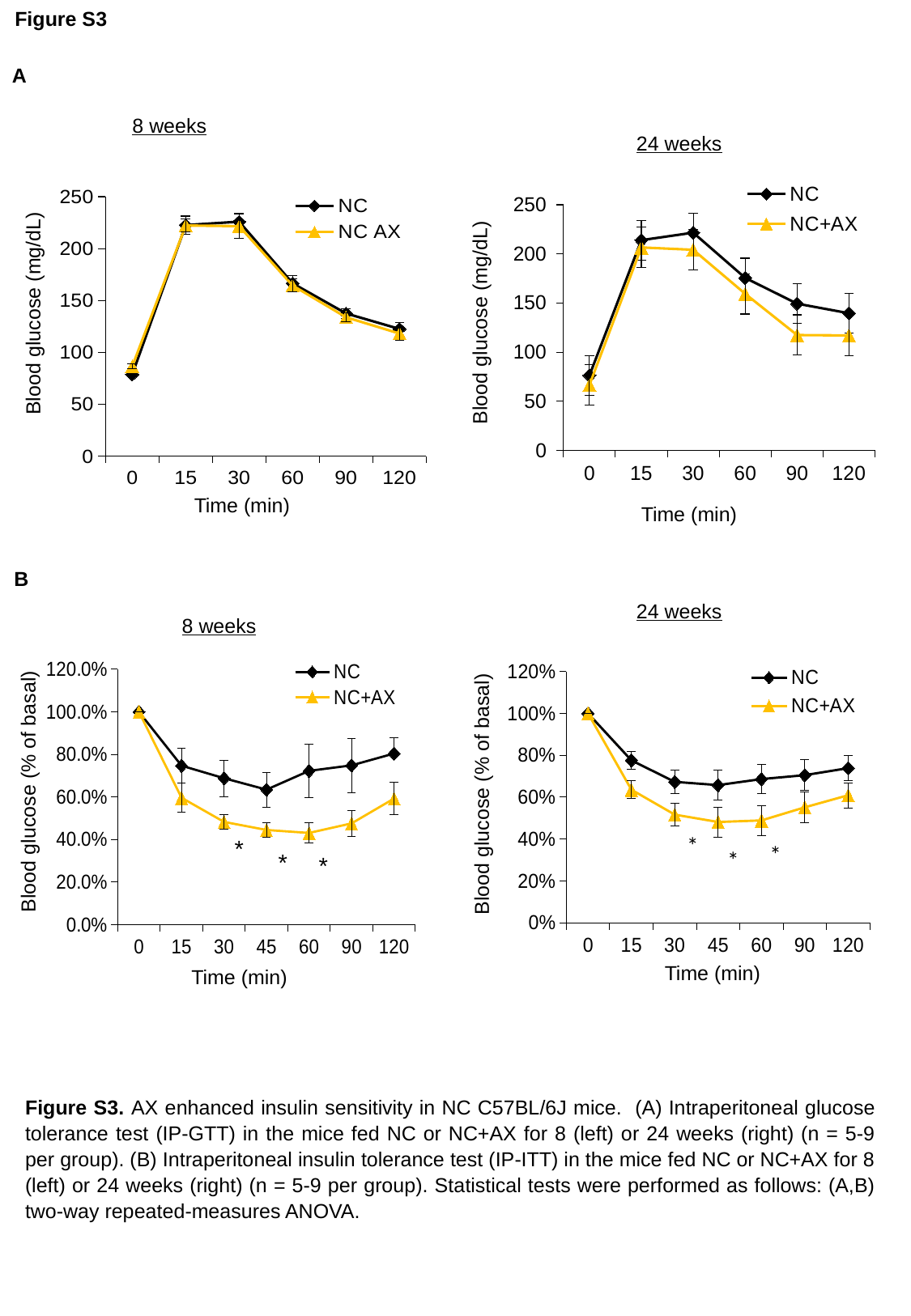

Figure S3
A
8 weeks
24 weeks
### Chart
| Category | NC | NC+AX |
|---|---|---|
| 0 | 76.3 | 66.6 |
| 15 | 214.0 | 206.6 |
| 30 | 221.6 | 204.2 |
| 60 | 175.6 | 159.4 |
| 90 | 149.4 | 117.4 |
| 120 | 139.6 | 117.0 |
### Chart
| Category | NC | NC AX |
|---|---|---|
| 0 | 78.83333333333333 | 86.57142857142857 |
| 15 | 222.66666666666666 | 222.21428571428572 |
| 30 | 225.94444444444446 | 221.57142857142858 |
| 60 | 166.55555555555554 | 164.42857142857142 |
| 90 | 137.61111111111111 | 133.92857142857142 |
| 120 | 122.61111111111111 | 118.21428571428571 |Blood glucose (mg/dL)
Blood glucose (mg/dL)
Time (min)
Time (min)
B
24 weeks
8 weeks
### Chart
| Category | | |
|---|---|---|
| 0 | 1.0 | 1.0 |
| 15 | 0.746900523914266 | 0.5962922690722618 |
| 30 | 0.6879957787885905 | 0.4825991853074935 |
| 45 | 0.6344620268933165 | 0.4444911304119552 |
| 60 | 0.7227402638079171 | 0.43066746017895796 |
| 90 | 0.748404980032887 | 0.4755553467365316 |
| 120 | 0.8043416547433461 | 0.5925350182045442 |
### Chart
| Category | | |
|---|---|---|
| 0 | 1.0 | 1.0 |
| 15 | 0.7758113217861635 | 0.635719179372769 |
| 30 | 0.673142985989815 | 0.5163367546567933 |
| 45 | 0.657672630274313 | 0.48122950659087754 |
| 60 | 0.6864297320849998 | 0.4886558562401978 |
| 90 | 0.705010059008982 | 0.5514941389797114 |
| 120 | 0.7387587197862283 | 0.6093438467065585 |Blood glucose (% of basal)
Blood glucose (% of basal)
*
*
*
*
*
*
Time (min)
Time (min)
Figure S3. AX enhanced insulin sensitivity in NC C57BL/6J mice. (A) Intraperitoneal glucose tolerance test (IP-GTT) in the mice fed NC or NC+AX for 8 (left) or 24 weeks (right) (n = 5-9 per group). (B) Intraperitoneal insulin tolerance test (IP-ITT) in the mice fed NC or NC+AX for 8 (left) or 24 weeks (right) (n = 5-9 per group). Statistical tests were performed as follows: (A,B) two-way repeated-measures ANOVA.

## Slide 6
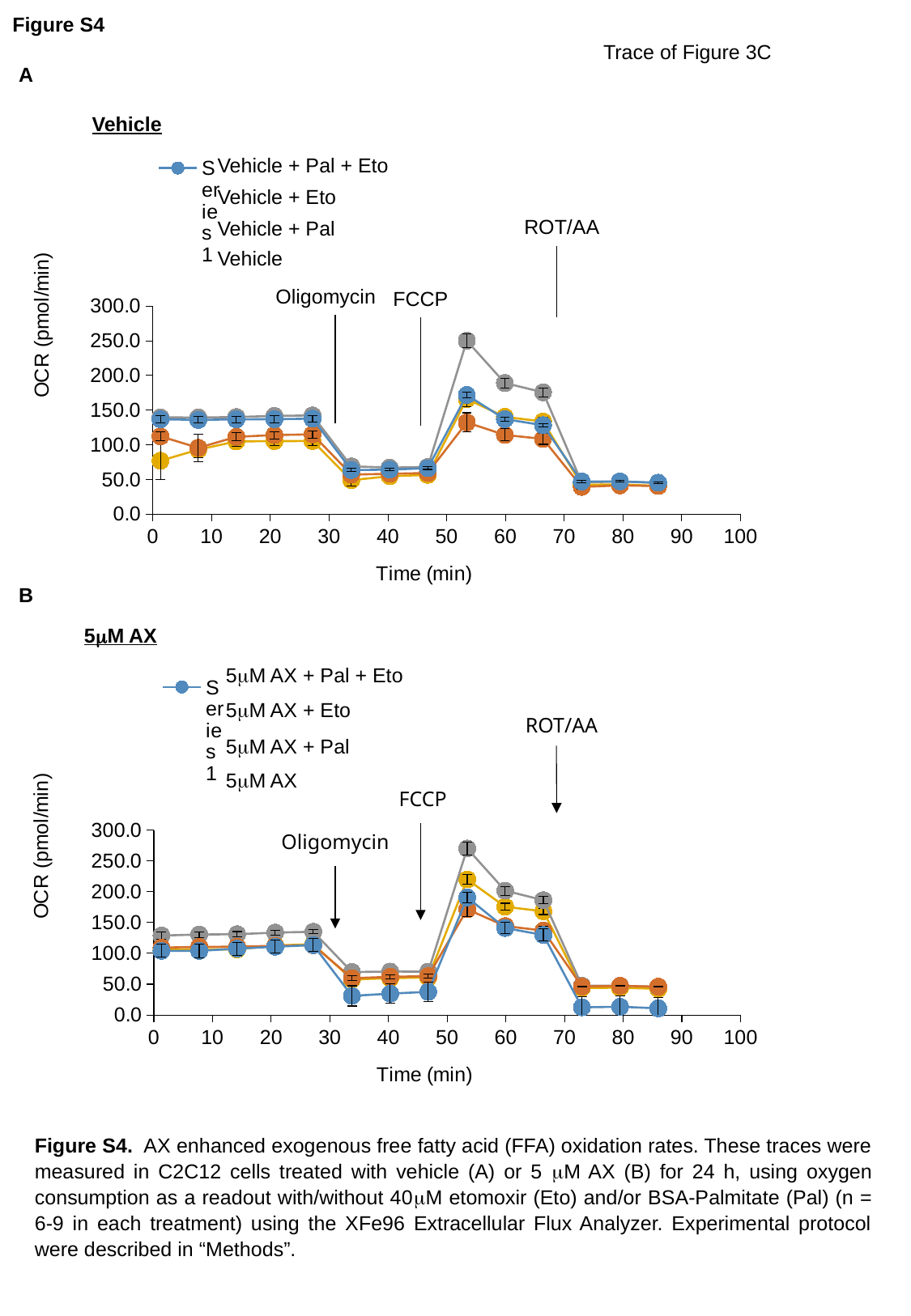

Figure S4
Trace of Figure 3C
A
Vehicle
### Chart
| Category | | | | | 10uM Met -/- | 10uM Met -/+ | 10uM Met +/- | 10uM Met +/+ | 5uM AX -/- | 5uM AX -/+ | 5uM AX +/- | 5uM AX +/+ |
|---|---|---|---|---|---|---|---|---|---|---|---|---|Vehicle + Pal + Eto
Vehicle + Eto
ROT/AA
Vehicle + Pal
Vehicle
Oligomycin
FCCP
B
5mM AX
5mM AX + Pal + Eto
### Chart
| Category | | | | | 10uM Met -/- | 10uM Met -/+ | 10uM Met +/- | 10uM Met +/+ | Vehicle -/- | Vehicle -/+ | Vehicle +/- | Vehicle +/+ |
|---|---|---|---|---|---|---|---|---|---|---|---|---|5mM AX + Eto
ROT/AA
5mM AX + Pal
5mM AX
FCCP
Oligomycin
Figure S4. AX enhanced exogenous free fatty acid (FFA) oxidation rates. These traces were measured in C2C12 cells treated with vehicle (A) or 5 mM AX (B) for 24 h, using oxygen consumption as a readout with/without 40mM etomoxir (Eto) and/or BSA-Palmitate (Pal) (n = 6-9 in each treatment) using the XFe96 Extracellular Flux Analyzer. Experimental protocol were described in “Methods”.

## Slide 7
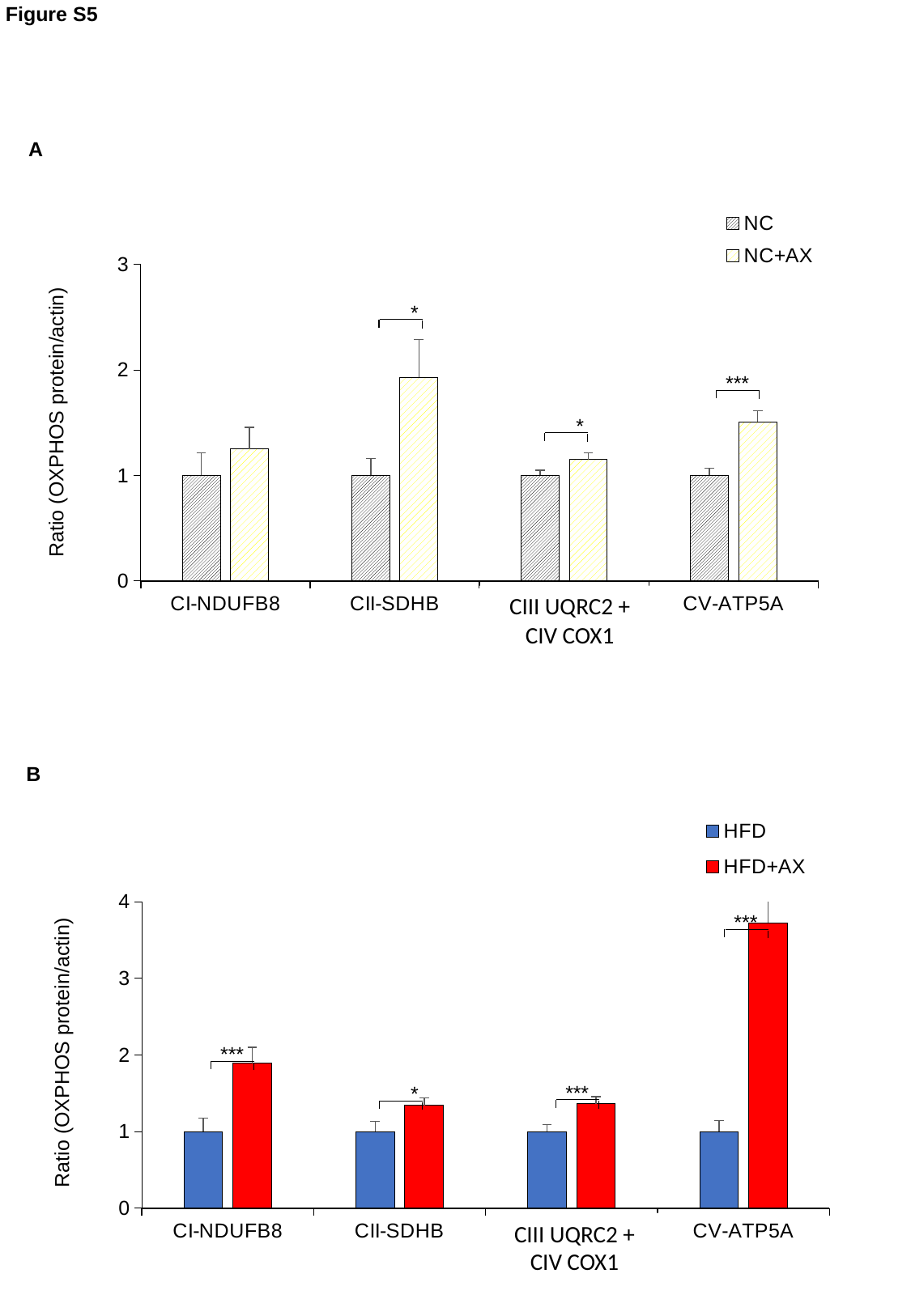

Figure S5
### Chart
| Category | NC | NC+AX |
|---|---|---|
| CI-NDUFB8 | 1.0 | 1.2502232646575233 |
| CII-SDHB | 0.9999999999999999 | 1.9310239079355735 |
| CIII-UQRC2 + CIV-MTCO1 | 1.0000000000000002 | 1.15086515003532 |
| CV-ATP5A | 1.0 | 1.5085697488927217 |A
*
***
Ratio (OXPHOS protein/actin)
*
CIII UQRC2 +
CIV COX1
### Chart
| Category | HFD | HFD+AX |
|---|---|---|
| CI-NDUFB8 | 1.0000000000000002 | 1.8884406102623148 |
| CII-SDHB | 0.9999999999999999 | 1.343978419328777 |
| CIII-UQRC2 + CIV-MTCO1 | 0.9999999999999997 | 1.365224738430613 |
| CV-ATP5A | 1.0 | 3.725253237943562 |B
***
Ratio (OXPHOS protein/actin)
***
***
*
CIII UQRC2 +
CIV COX1

## Slide 8
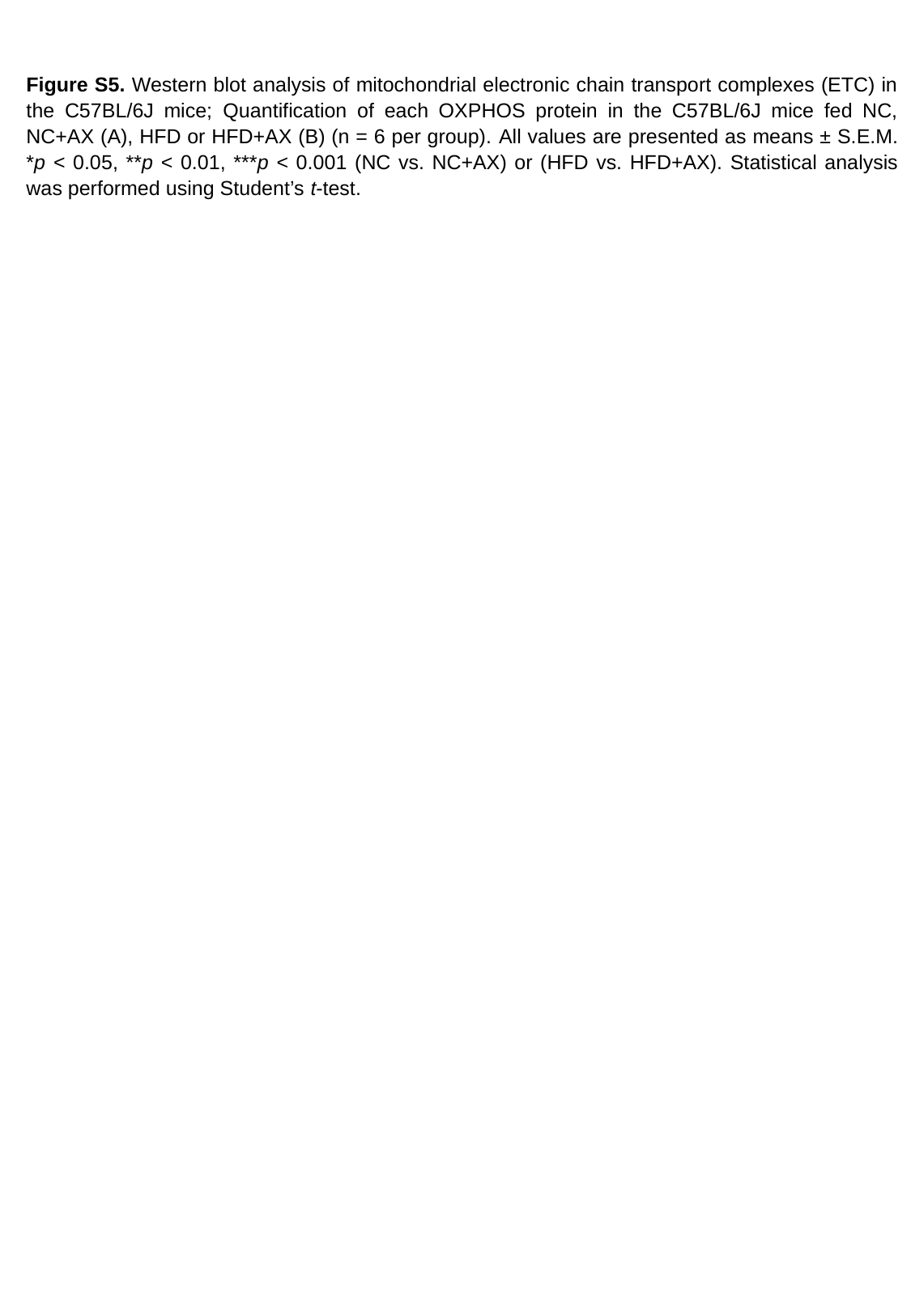

Figure S5. Western blot analysis of mitochondrial electronic chain transport complexes (ETC) in the C57BL/6J mice; Quantification of each OXPHOS protein in the C57BL/6J mice fed NC, NC+AX (A), HFD or HFD+AX (B) (n = 6 per group). All values are presented as means ± S.E.M. *p < 0.05, **p < 0.01, ***p < 0.001 (NC vs. NC+AX) or (HFD vs. HFD+AX). Statistical analysis was performed using Student’s t-test.

## Slide 9
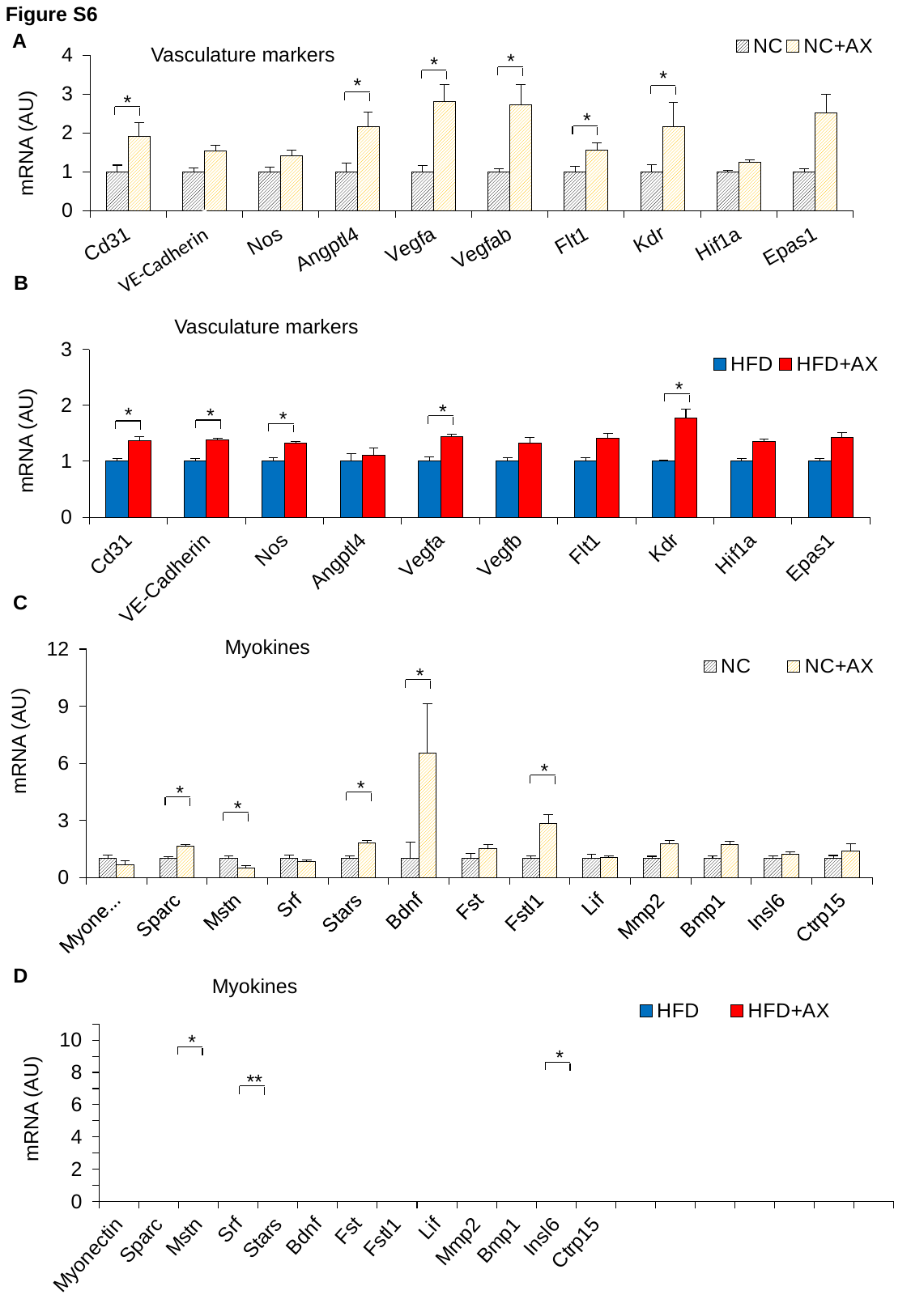

Figure S6
### Chart
| Category | NC | NC+AX |
|---|---|---|
| Cd31 | 1.0 | 1.9157142747967757 |
| VE-cadherin | 1.0 | 1.5299403349692817 |
| Nos | 0.9999999999999998 | 1.4021779123198164 |
| Angptl4 | 1.0 | 2.1615596871769682 |
| Vegfa | 1.0 | 2.7941039287450042 |
| Vegfab | 1.0 | 2.7166451263701954 |
| Flt1 | 1.0 | 1.5579227298573428 |
| Kdr | 1.0 | 2.1667668279082015 |
| Hif1a | 1.0000000000000002 | 1.246826702792135 |
| Epas1 | 1.0 | 2.5027402002681605 |A
Vasculature markers
*
*
*
*
*
*
mRNA (AU)
VE-Cadherin
B
Vasculature markers
### Chart
| Category | HFD | HFD+AX |
|---|---|---|
| Cd31 | 1.0 | 1.365819257179951 |
| VE-Cadherin | 1.0000000000000002 | 1.3756268988439093 |
| Nos | 1.0 | 1.3184562282586423 |
| Angptl4 | 0.9999999999999999 | 1.114375463879401 |
| Vegfa | 1.0 | 1.4462148683294107 |
| Vegfb | 0.9999999999999999 | 1.3175959395367818 |
| Flt1 | 1.0 | 1.4048122382398958 |
| Kdr | 1.0000000000000002 | 1.7720760865252936 |
| Hif1a | 1.0 | 1.3552519038539572 |
| Epas1 | 0.9999999999999999 | 1.4195351204067421 |*
*
*
*
*
mRNA (AU)
C
Myokines
### Chart
| Category | NC | NC+AX |
|---|---|---|
| Myonectin | 1.0000000000000002 | 0.6792690452051656 |
| Sparc | 1.0 | 1.6555625452969023 |
| Mstn | 1.0 | 0.5 |
| Srf | 0.9999999999999999 | 0.8182108882049954 |
| Stars | 1.0 | 1.7957933929953416 |
| Bdnf | 1.0 | 6.5459516710885906 |
| Fst | 1.0 | 1.5165675586667187 |
| Fstl1 | 1.0 | 2.82785310079599 |
| Lif | 1.0 | 1.0335879131155925 |
| Mmp2 | 1.0 | 1.7702605802842968 |
| Bmp1 | 1.0 | 1.7167288687615596 |
| Insl6 | 1.0 | 1.2292994864334508 |
| Ctrp15 | 1.0 | 1.3993892045934608 |*
mRNA (AU)
*
*
*
*
D
Myokines
### Chart
| Category | | |
|---|---|---|
| Myonectin | 1.0 | 1.0246263633771433 |
| Sparc | 0.9999999999999997 | 1.4075096095771993 |
| Mstn | 1.0000000000000002 | 0.5348007875411406 |
| Srf | 1.0000000000000002 | 1.0167040580830342 |
| Stars | 0.9999999999999998 | 1.3080160790366195 |
| Bdnf | 1.0 | 1.226986388574639 |
| Fst | 1.0000000000000002 | 1.1219082004823322 |
| Fstl1 | 1.0000000000000002 | 1.2729785571231336 |
| Lif | 1.0000000000000002 | 1.2591136299852062 |
| Mmp2 | 1.0 | 0.9933354992107756 |
| Bmp1 | 1.0 | 1.1838812813418906 |
| Insl6 | 1.0 | 0.8719518179394631 |
| Ctrp15 | 1.0 | 1.0928610010108297 |*
*
**
mRNA (AU)

## Slide 10
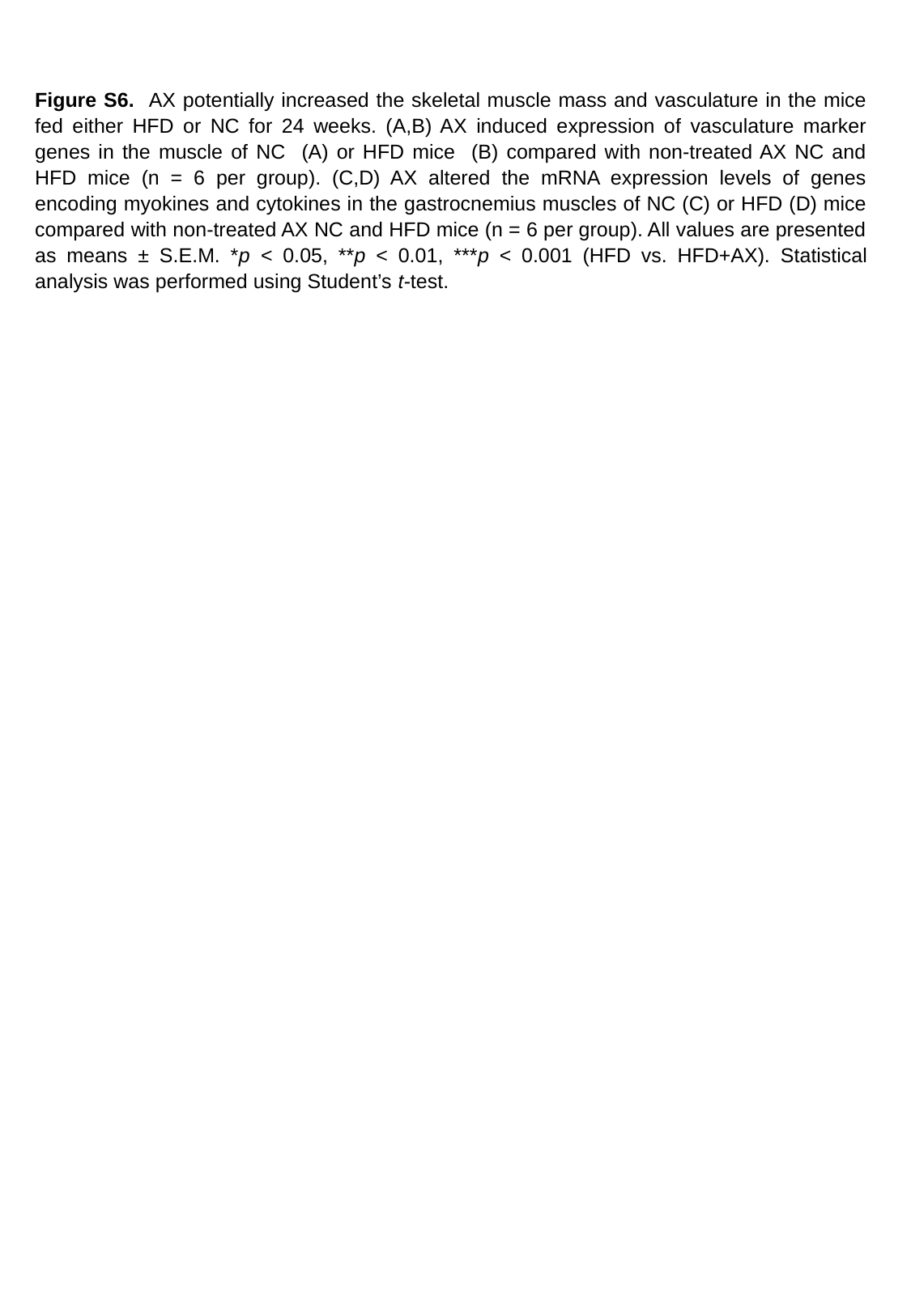

Figure S6. AX potentially increased the skeletal muscle mass and vasculature in the mice fed either HFD or NC for 24 weeks. (A,B) AX induced expression of vasculature marker genes in the muscle of NC (A) or HFD mice (B) compared with non-treated AX NC and HFD mice (n = 6 per group). (C,D) AX altered the mRNA expression levels of genes encoding myokines and cytokines in the gastrocnemius muscles of NC (C) or HFD (D) mice compared with non-treated AX NC and HFD mice (n = 6 per group). All values are presented as means ± S.E.M. *p < 0.05, **p < 0.01, ***p < 0.001 (HFD vs. HFD+AX). Statistical analysis was performed using Student’s t-test.

## Slide 11
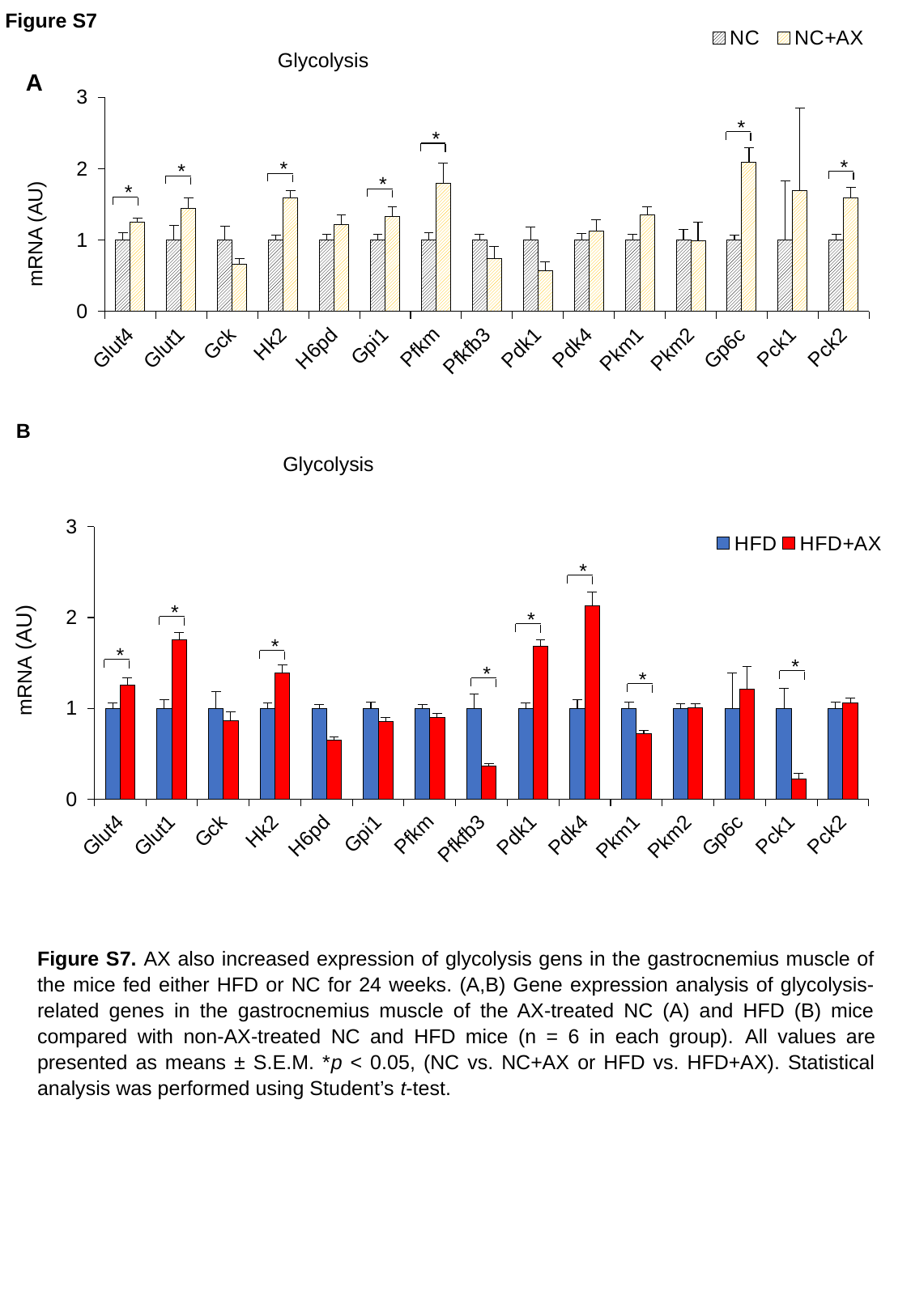

### Chart
| Category | NC | NC+AX |
|---|---|---|
| Glut4 | 1.0000000000000002 | 1.2494586664163847 |
| Glut1 | 1.0 | 1.4436136761094194 |
| Gck | 1.0 | 0.65665087098214 |
| Hk2 | 0.9999999999999997 | 1.589015066640522 |
| H6pd | 1.0 | 1.217728679430493 |
| Gpi1 | 1.0 | 1.335322946484222 |
| Pfkm | 1.0 | 1.7989392966841091 |
| Pfkfb3 | 1.0 | 0.7413732648687729 |
| Pdk1 | 0.9999999999999999 | 0.5768933480152587 |
| Pdk4 | 1.0000000000000002 | 1.13010776741745 |
| Pkm1 | 1.0 | 1.3543395909047742 |
| Pkm2 | 1.0000000000000002 | 0.9918040556728283 |
| Gp6c | 1.0000000000000002 | 2.095027378780824 |
| Pck1 | 1.0 | 1.6894698249985707 |
| Pck2 | 1.0 | 1.5922470975352507 |Figure S7
Glycolysis
A
*
*
*
*
*
*
*
mRNA (AU)
B
Glycolysis
### Chart
| Category | HFD | HFD+AX |
|---|---|---|
| Glut4 | 0.9999999999999999 | 1.2583676165173625 |
| Glut1 | 1.0 | 1.7515065987188763 |
| Gck | 1.0 | 0.8621909314130397 |
| Hk2 | 0.9999999999999999 | 1.3866898076502616 |
| H6pd | 1.0 | 0.6521305273027094 |
| Gpi1 | 1.0 | 0.8540099984652563 |
| Pfkm | 1.0000000000000002 | 0.8950309739413697 |
| Pfkfb3 | 1.0 | 0.3615343105346487 |
| Pdk1 | 1.0 | 1.687328377455309 |
| Pdk4 | 1.0000000000000002 | 2.1301077674174524 |
| Pkm1 | 1.0000000000000002 | 0.7200166891912089 |
| Pkm2 | 0.9999999999999999 | 1.007415639702265 |
| Gp6c | 0.9999999999999999 | 1.2145085730236997 |
| Pck1 | 1.0000000000000002 | 0.2215947412242456 |
| Pck2 | 1.0000000000000002 | 1.059329547292741 |*
*
*
*
*
mRNA (AU)
*
*
*
Figure S7. AX also increased expression of glycolysis gens in the gastrocnemius muscle of the mice fed either HFD or NC for 24 weeks. (A,B) Gene expression analysis of glycolysis-related genes in the gastrocnemius muscle of the AX-treated NC (A) and HFD (B) mice compared with non-AX-treated NC and HFD mice (n = 6 in each group). All values are presented as means ± S.E.M. *p < 0.05, (NC vs. NC+AX or HFD vs. HFD+AX). Statistical analysis was performed using Student’s t-test.

## Slide 12
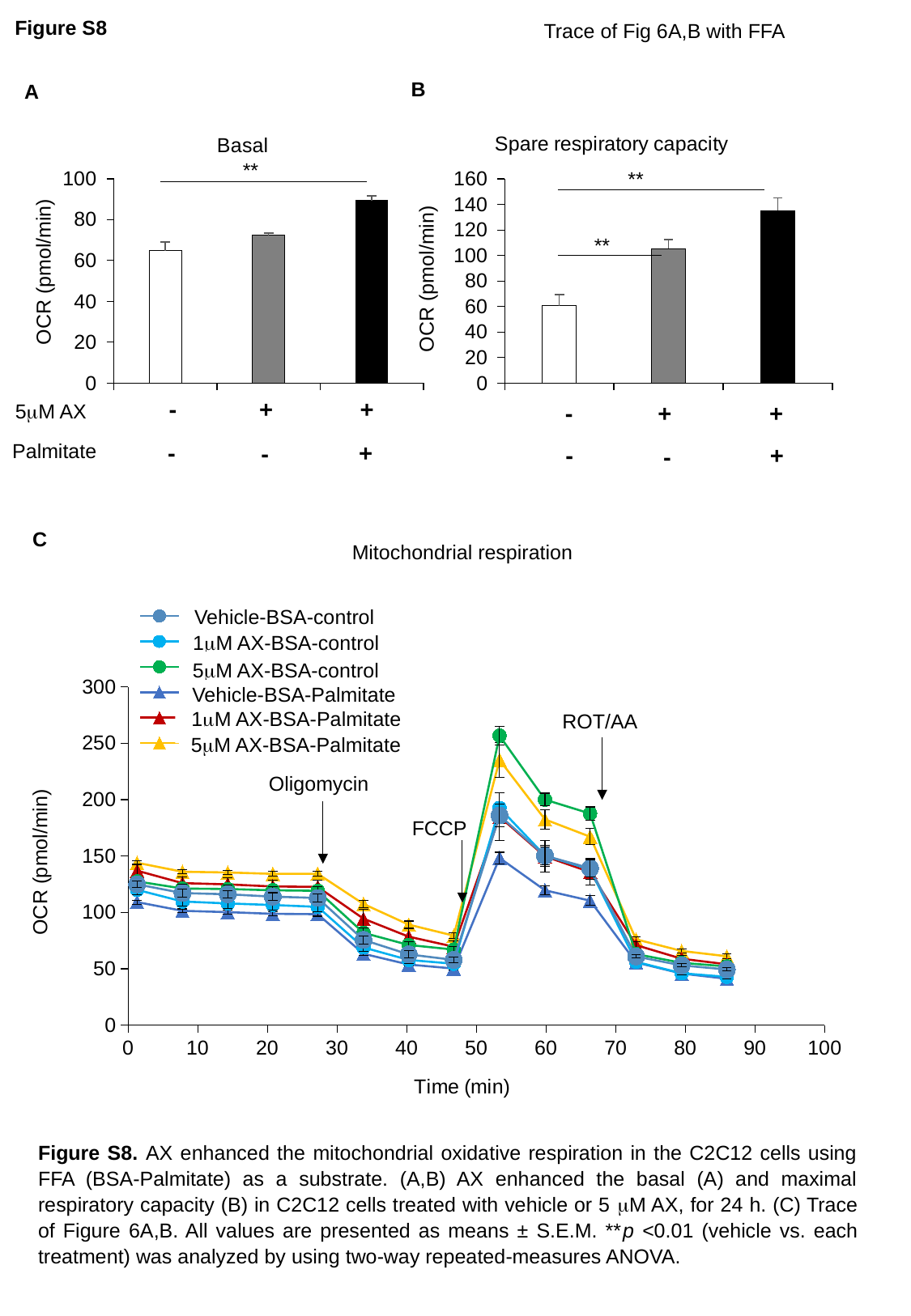

Figure S8
Trace of Fig 6A,B with FFA
B
A
### Chart:
| Category | Basal |
|---|---|
| Vehicle w/o FFA | 64.76792907714844 |
| Vehicle w/ FFA | 72.2789077758789 |
| 5uM AX w/ FFA | 89.39561462402344 |
### Chart: Spare respiratory capacity
| Category | Spare Respiratory Capacity |
|---|---|**
**
**
OCR (pmol/min)
OCR (pmol/min)
-
+
+
-
+
+
5mM AX
-
+
Palmitate
-
-
+
-
C
Mitochondrial respiration
### Chart
| Category | Vehicle - BSA-control | 5uM AX - BSA-control | 25uM AX - BSA-control | Vehicle - BSA-Palmitate | 5uM AX - BSA-Palmitate | 25uM AX - BSA-Palmitate |
|---|---|---|---|---|---|---|Vehicle-BSA-control
1mM AX-BSA-control
5mM AX-BSA-control
Vehicle-BSA-Palmitate
1mM AX-BSA-Palmitate
ROT/AA
1mM AX-BSA-Palmitate
5mM AX-BSA-Palmitate
Oligomycin
FCCP
Figure S8. AX enhanced the mitochondrial oxidative respiration in the C2C12 cells using FFA (BSA-Palmitate) as a substrate. (A,B) AX enhanced the basal (A) and maximal respiratory capacity (B) in C2C12 cells treated with vehicle or 5 mM AX, for 24 h. (C) Trace of Figure 6A,B. All values are presented as means ± S.E.M. **p <0.01 (vehicle vs. each treatment) was analyzed by using two-way repeated-measures ANOVA.

## Slide 13
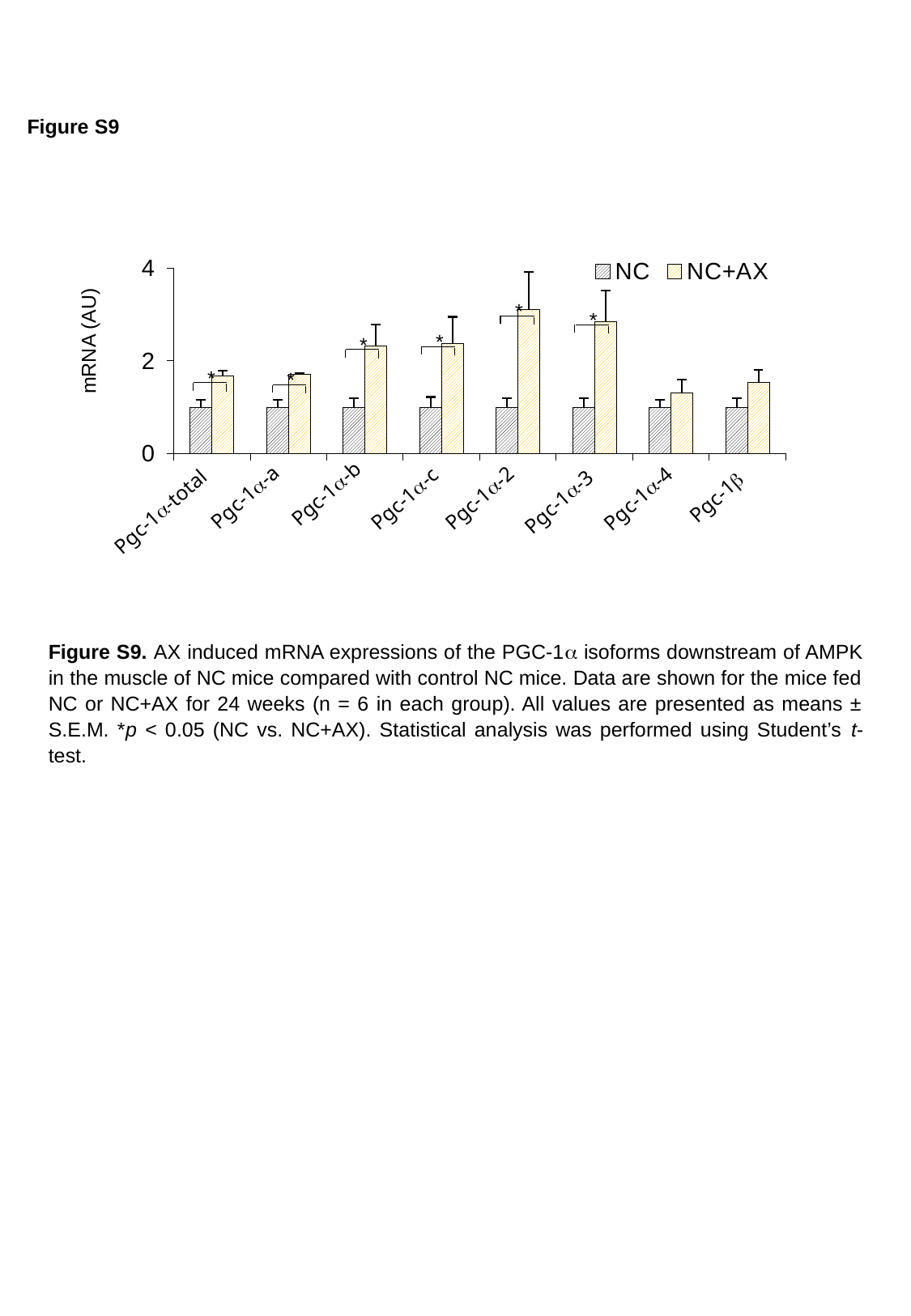

Figure S9
### Chart
| Category | NC | NC+AX |
|---|---|---|
| PGC1a-Total | 1.0000000000000002 | 1.680595519076056 |
| PGC1a-a | 1.0 | 1.7082659568697827 |
| PGC1a-b | 0.9999999999999997 | 2.3141154939346036 |
| PGC1a-c | 1.0000000000000002 | 2.3698575836967435 |
| PGC1a-2 | 1.0 | 3.1083078144352148 |
| PGC1a-3 | 0.9999999999999997 | 2.8382502724184047 |
| PGC1a-4 | 1.0000000000000002 | 1.3149688619461788 |
| PGC1b | 1.0 | 1.5285013864378527 |*
*
mRNA (AU)
*
*
*
*
Pgc-1a-b
Pgc-1a-a
Pgc-1b
Pgc-1a-2
Pgc-1a-4
Pgc-1a-c
Pgc-1a-3
Pgc-1a-total
Figure S9. AX induced mRNA expressions of the PGC-1a isoforms downstream of AMPK in the muscle of NC mice compared with control NC mice. Data are shown for the mice fed NC or NC+AX for 24 weeks (n = 6 in each group). All values are presented as means ± S.E.M. *p < 0.05 (NC vs. NC+AX). Statistical analysis was performed using Student’s t-test.

## Slide 14
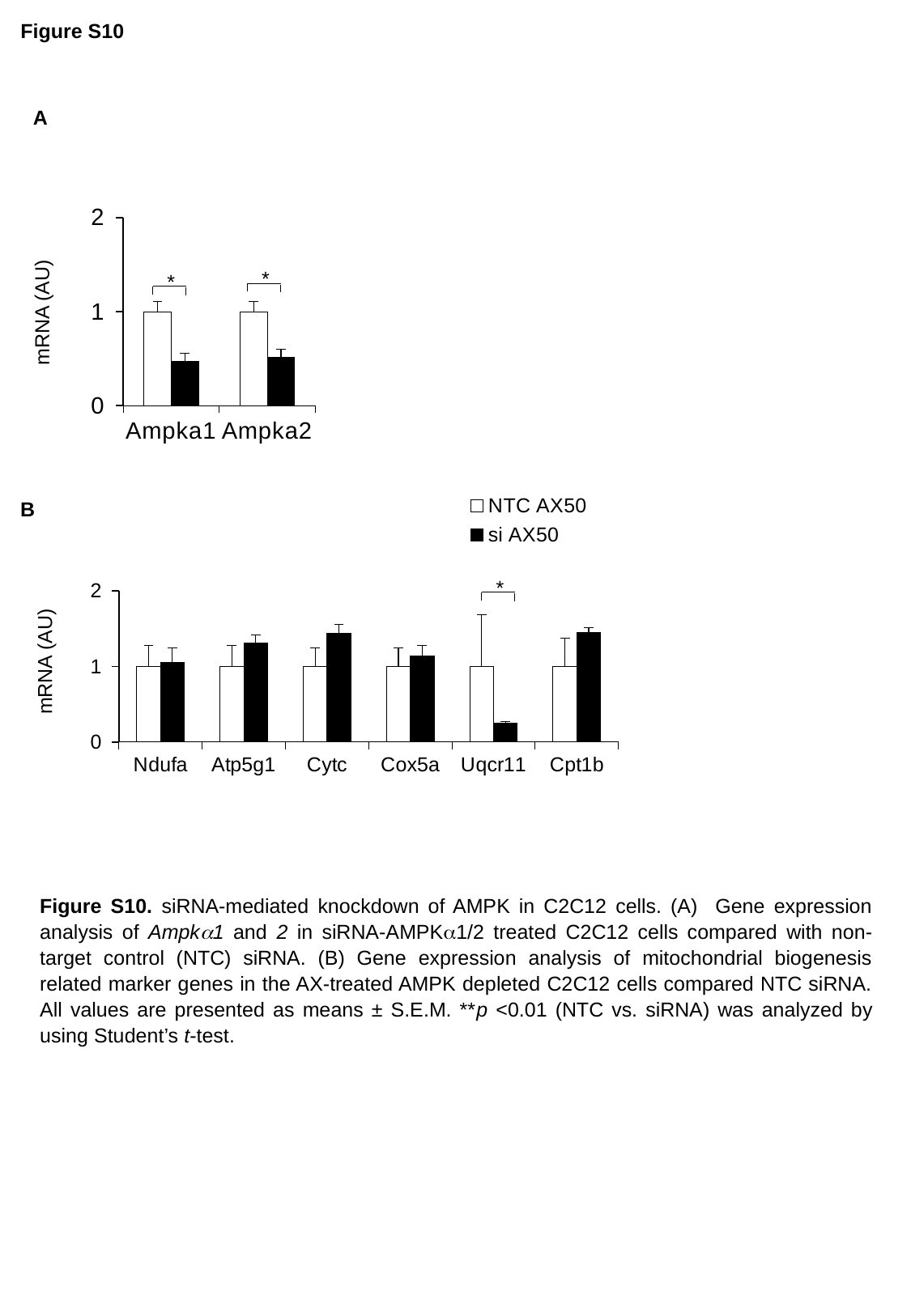

Figure S10
A
### Chart
| Category | NTC | siRNA |
|---|---|---|
| Ampka1 | 1.0 | 0.4706906623974529 |
| Ampka2 | 1.0 | 0.5167497733710497 |*
*
mRNA (AU)
### Chart
| Category | NTC AX50 | si AX50 |
|---|---|---|
| Ndufa | 1.0 | 1.0501171560764644 |
| Atp5g1 | 1.0 | 1.3051378246799876 |
| Cytc | 1.0 | 1.4433320494438062 |
| Cox5a | 1.0 | 1.1371134735536006 |
| Uqcr11 | 1.0 | 0.24944099092488312 |
| Cpt1b | 1.0 | 1.4462679425472595 |B
*
mRNA (AU)
Figure S10. siRNA-mediated knockdown of AMPK in C2C12 cells. (A) Gene expression analysis of Ampka1 and 2 in siRNA-AMPKa1/2 treated C2C12 cells compared with non-target control (NTC) siRNA. (B) Gene expression analysis of mitochondrial biogenesis related marker genes in the AX-treated AMPK depleted C2C12 cells compared NTC siRNA. All values are presented as means ± S.E.M. **p <0.01 (NTC vs. siRNA) was analyzed by using Student’s t-test.

## Slide 15
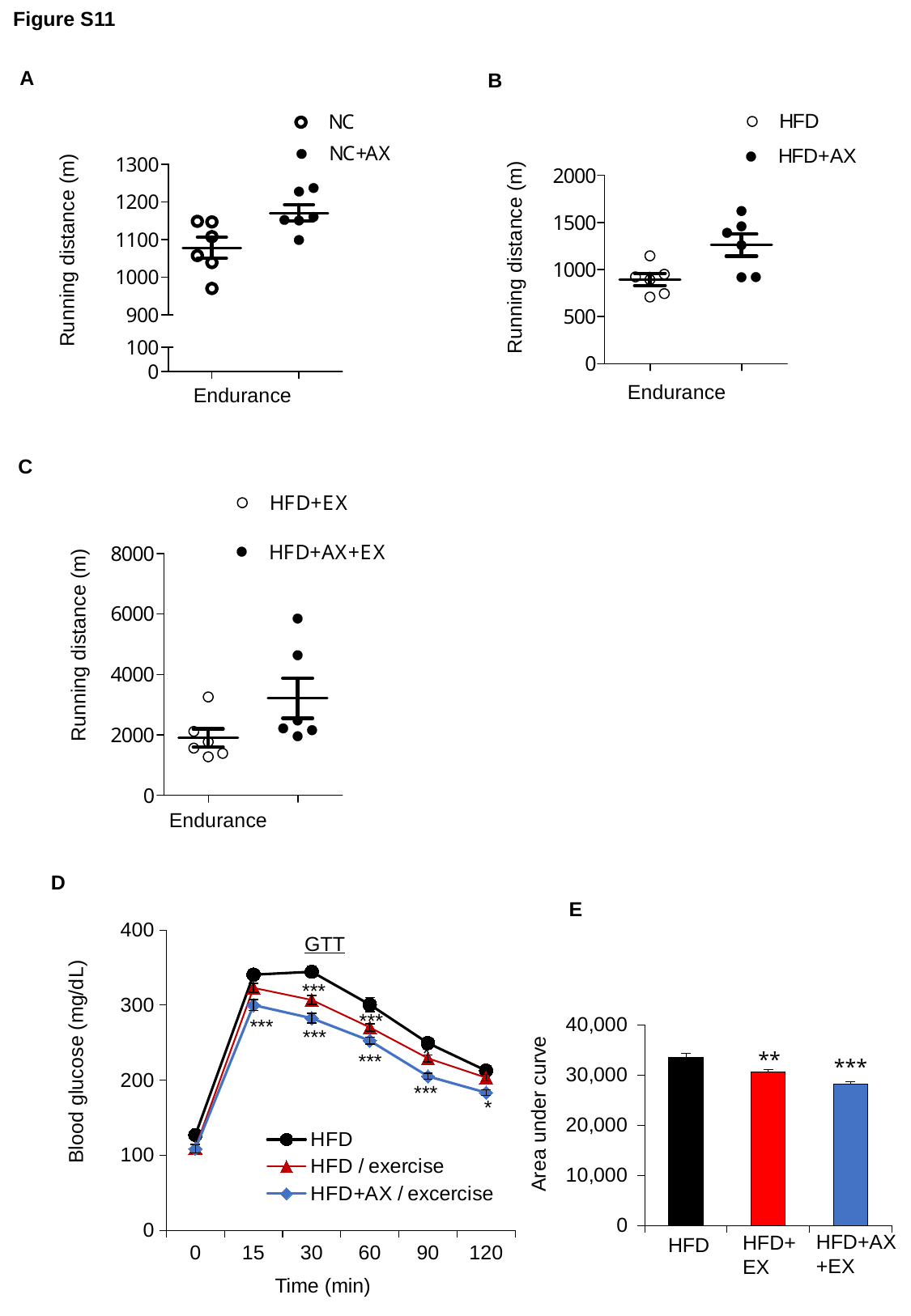

Figure S11
A
B
Running distance (m)
Running distance (m)
Endurance
Endurance
C
Running distance (m)
Endurance
D
E
### Chart
| Category | HFD |
|---|---|
| HFD | 33586.041666666664 |
| HFD+EX | 30616.041666666668 |
| HFD+AX+EX | 28155.208333333332 |
### Chart
| Category | HFD | HFD / exercise | HFD+AX / excercise |
|---|---|---|---|
| 0 | 126.97222222222221 | 110.06944444444446 | 108.52777777777777 |
| 15 | 340.6388888888889 | 323.0555555555556 | 300.0416666666667 |
| 30 | 344.5 | 306.9583333333333 | 282.5 |
| 60 | 300.7638888888889 | 270.4166666666667 | 252.63888888888889 |
| 90 | 249.41666666666666 | 229.16666666666666 | 205.125 |
| 120 | 212.70833333333334 | 203.375 | 183.43055555555554 |GTT
***
***
***
***
*
***
***
*
**
Blood glucose (mg/dL)
***
Area under curve
HFD+AX+EX
HFD+EX
HFD
Time (min)

## Slide 16
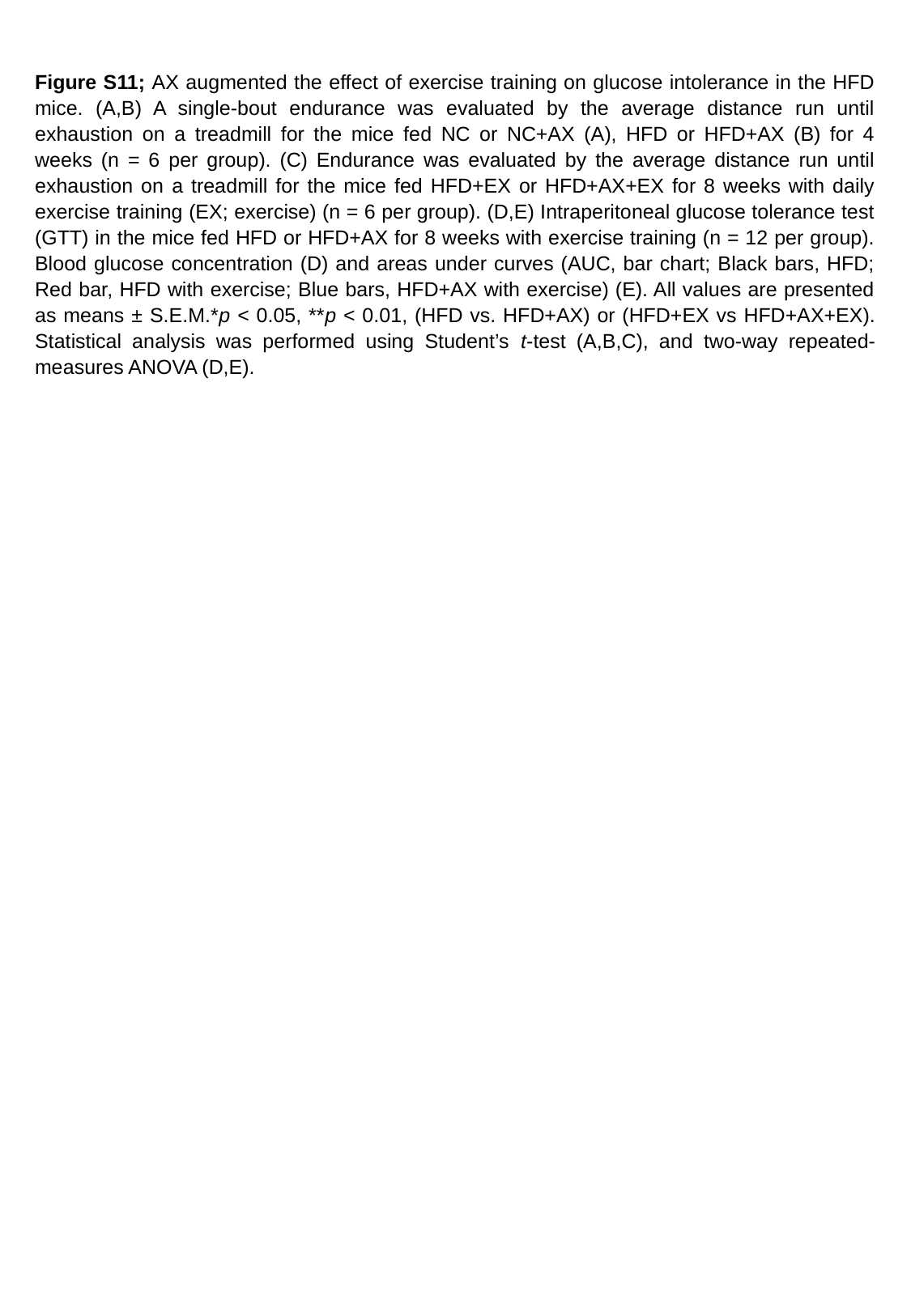

Figure S11; AX augmented the effect of exercise training on glucose intolerance in the HFD mice. (A,B) A single-bout endurance was evaluated by the average distance run until exhaustion on a treadmill for the mice fed NC or NC+AX (A), HFD or HFD+AX (B) for 4 weeks (n = 6 per group). (C) Endurance was evaluated by the average distance run until exhaustion on a treadmill for the mice fed HFD+EX or HFD+AX+EX for 8 weeks with daily exercise training (EX; exercise) (n = 6 per group). (D,E) Intraperitoneal glucose tolerance test (GTT) in the mice fed HFD or HFD+AX for 8 weeks with exercise training (n = 12 per group). Blood glucose concentration (D) and areas under curves (AUC, bar chart; Black bars, HFD; Red bar, HFD with exercise; Blue bars, HFD+AX with exercise) (E). All values are presented as means ± S.E.M.*p < 0.05, **p < 0.01, (HFD vs. HFD+AX) or (HFD+EX vs HFD+AX+EX). Statistical analysis was performed using Student’s t-test (A,B,C), and two-way repeated-measures ANOVA (D,E).

## Slide 17
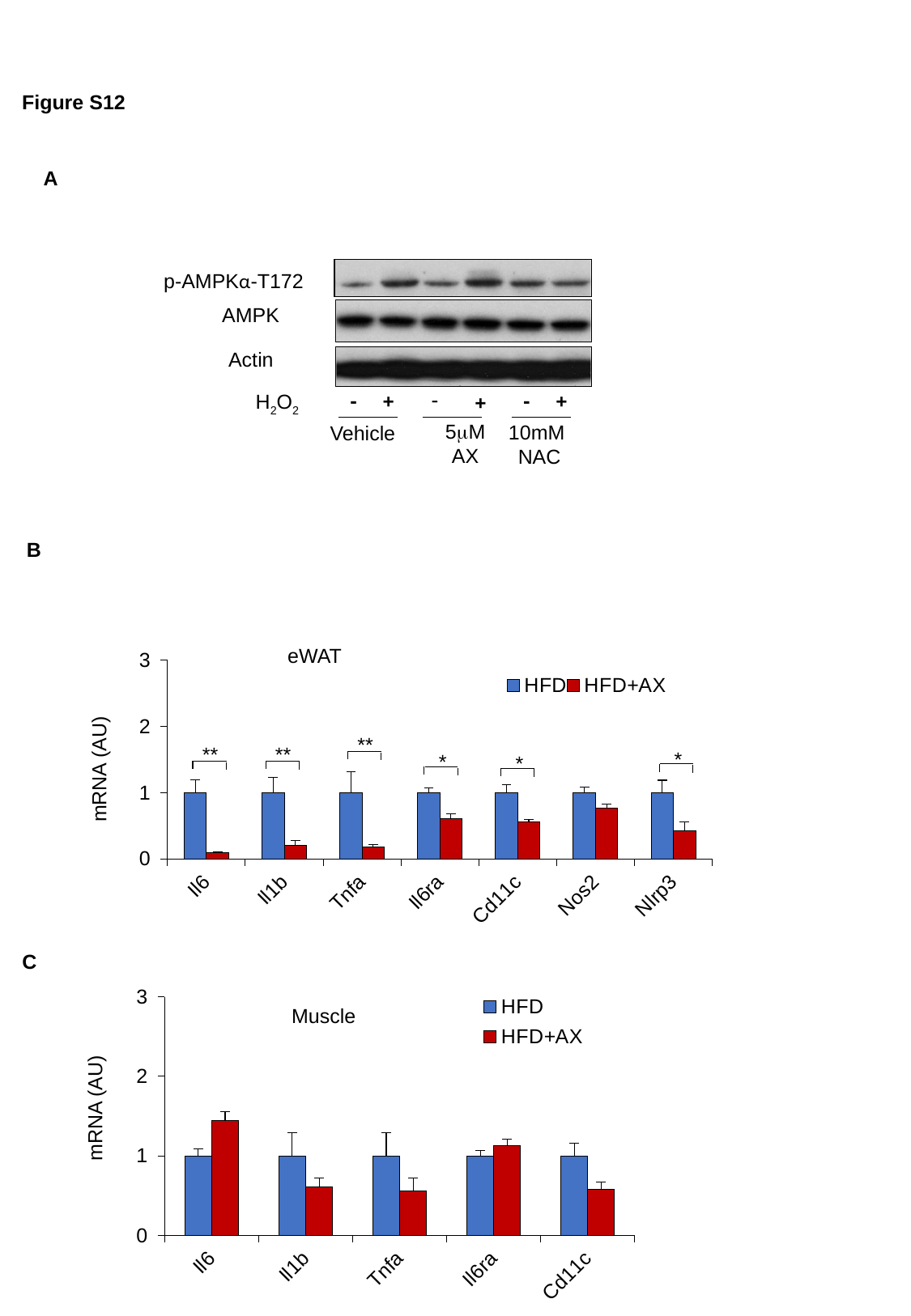

Figure S12
A
p-AMPKα-T172
AMPK
Actin
-
-
-
+
+
H2O2
+
5mM
AX
10mM
NAC
Vehicle
B
eWAT
### Chart
| Category | HFD | HFD+AX |
|---|---|---|
| Il6 | 1.0000000000000002 | 0.09409191294607094 |
| Il1b | 1.0 | 0.1998183549874685 |
| Tnfa | 1.0 | 0.18375834397882337 |
| Il6ra | 1.0 | 0.6118516787181446 |
| Cd11c | 0.9999999999999999 | 0.5536037141109588 |
| Nos2 | 0.9999999999999999 | 0.76486548964137 |
| Nlrp3 | 1.0000000000000002 | 0.42033944080305946 |**
**
**
*
*
*
mRNA (AU)
C
### Chart
| Category | HFD | HFD+AX |
|---|---|---|
| Il6 | 1.0 | 1.4480289763960412 |
| Il1b | 0.9999999999999999 | 0.6080530685640173 |
| Tnfa | 1.0 | 0.5564165277948458 |
| Il6ra | 1.0 | 1.1344689272655113 |
| Cd11c | 1.0 | 0.5847068907958204 |Muscle
mRNA (AU)

## Slide 18
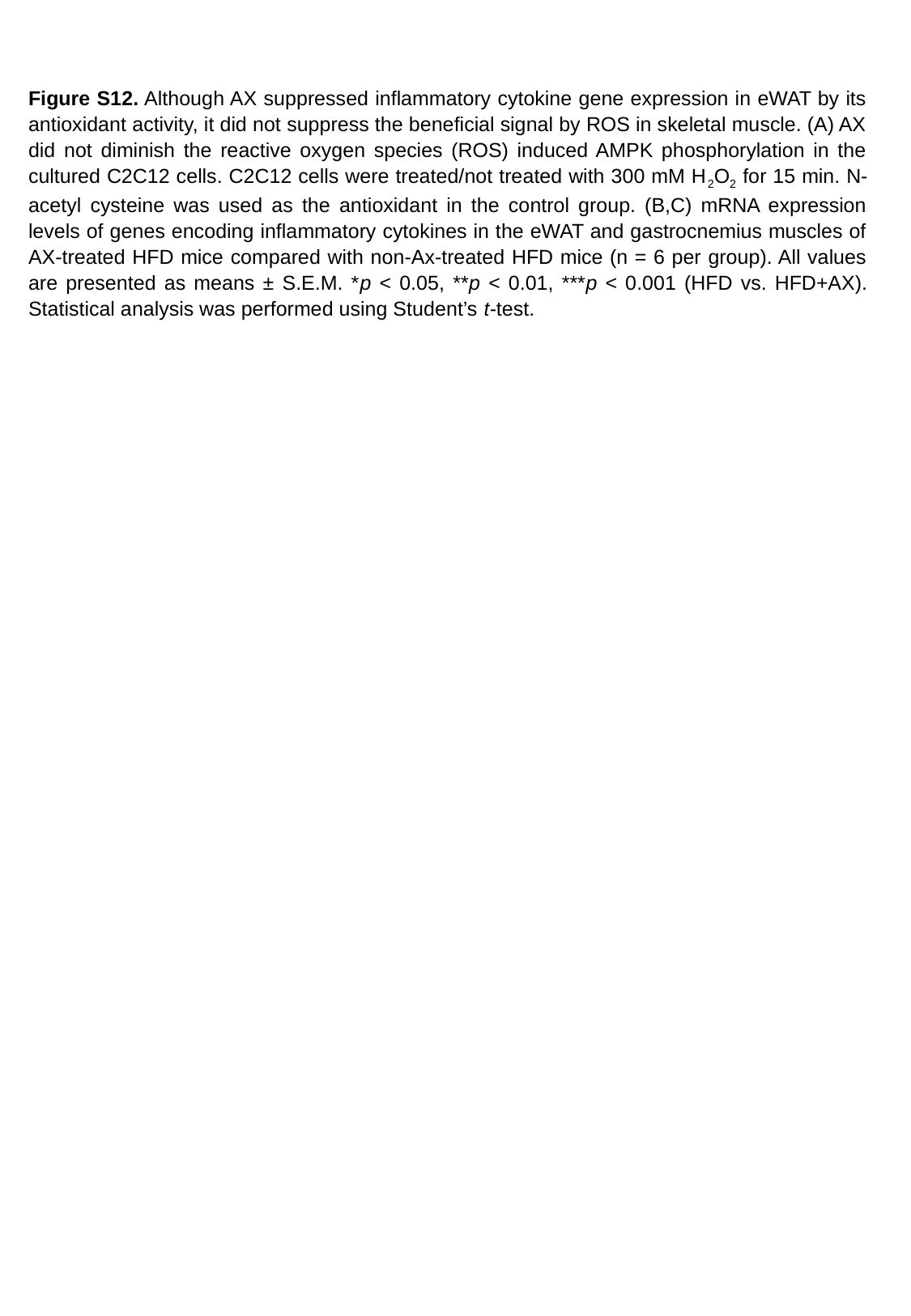

Figure S12. Although AX suppressed inflammatory cytokine gene expression in eWAT by its antioxidant activity, it did not suppress the beneficial signal by ROS in skeletal muscle. (A) AX did not diminish the reactive oxygen species (ROS) induced AMPK phosphorylation in the cultured C2C12 cells. C2C12 cells were treated/not treated with 300 mM H2O2 for 15 min. N-acetyl cysteine was used as the antioxidant in the control group. (B,C) mRNA expression levels of genes encoding inflammatory cytokines in the eWAT and gastrocnemius muscles of AX-treated HFD mice compared with non-Ax-treated HFD mice (n = 6 per group). All values are presented as means ± S.E.M. *p < 0.05, **p < 0.01, ***p < 0.001 (HFD vs. HFD+AX). Statistical analysis was performed using Student’s t-test.
